# STR-GNN: stability-regularized graph neural networks for suppressing spurious temporal variations in dynamic community detection

**DOI:** 10.1038/s41598-026-54851-z

**Published:** 2026-06-01

**Authors:** Daozheng Qu, Yanfei Ma, Yibo Wang

**Affiliations:** 1 Department of Computer Science, Fairleigh Dickinson University, V6B 2P6 Vancouver, Canada; 2 Department of Computer Science, University of Liverpool, L69 3DR Liverpool, UK; 3 Rady School of Management, University of California San Diego, 92093 San Diego, USA

**Keywords:** Dynamic networks, Community evolution, Uncertainty-aware learning, Temporal graph analysis, Robust community detection, Bayesian modeling, Temporal stability, Mathematics and computing, Neuroscience, Physics, Psychology, Psychology

## Abstract

The study of temporal graphs frequently encounters spurious temporal fluctuations, wherein transient noise, inadequate observations, or ephemeral structural perturbations result in unstable and inconsistent community assignments. In dynamic networks, such variations may stem from measurement inaccuracies, sampling biases, or sudden yet non-informative changes in topology, leading graph neural networks (GNNs) to excessively respond to local fluctuations rather than accurately reflecting the genuine growth of communities. Consequently, the acquired representations may demonstrate considerable temporal inconsistency, resulting in community structures that fluctuate erratically across successive time intervals. This instability significantly constrains the reliability of dynamic community recognition in temporal GNNs, especially in streaming, partially observed, or weakly supervised contexts, where the model must perpetually adjust to changing graph structures without comprehensive ground-truth supervision. In these circumstances, the learning process may provide community assignments that seem credible at each moment yet lack overall temporal consistency. We refer to this issue as spurious temporal variation, where temporal GNNs produce unstable community assignments caused by transient perturbations rather than genuine structural evolution. The suggested method mitigates false temporal fluctuations by implementing stability-aware regularization and consistency restrictions across successive graph snapshots, promoting the model’s ability to maintain coherent community structures while adapting to authentic structural changes. The proposed framework enhances the resilience, reliability, and interpretability of temporal GNN-based community identification by stabilizing community evolution over time, hence rendering it more appropriate for real-world dynamic networks characterized by noise, perturbations, and incomplete data.

## Introduction

The goal of dynamic community detection is to recognize evolving group structures in temporal graphs. This is because the interactions between nodes and the patterns of connectivity between them change over time as a result of intrinsic structural drift, random disturbances, and incomplete observations. In real-world dynamic networks, such as social systems, communication networks, and biological interactions, community structures almost never evolve in a smooth^1^. Instead, they frequently display erratic fluctuations that are brought on by noise, sampling bias, or brief structural disturbances. As a result, it is a difficult task to accurately trace the evolution of the community. Therefore, in order to achieve strong dynamic community detection, it is vital to accurately differentiate between true structural changes and misleading variations^2^.

Temporal graph neural networks, also known as TGNNs, have lately emerged as a powerful paradigm for modeling time-dependent interactions. This is due to the fact that TGNNs are able to acquire expressive node representations that combine both spatial topology and temporal dependencies^3^. TGNN-based approaches are able to capture the evolution of node associations and give excellent embeddings for downstream community recognition. This is accomplished by aggregating information across consecutive graph snapshots. As a result, TGNNs continue to be extremely sensitive to perturbations at the snapshot level, despite the fact that they have superior representational capabilities. During the process of temporal propagation, it is possible for little structural noise, missing edges, or interactions that are only temporary to be amplified^4^. This can cause the model to overfit transient patterns rather than the genuine underlying evolution of the graph. As a consequence, learned representations may generate unstable community assignments over time, including artificial splits, merges, or oscillatory membership changes. We use the term spurious temporal fluctuations to describe such unstable community changes caused by transient perturbations rather than true structural evolution^5^. This describes a situation in which apparent community changes are produced by model instability rather than true structural evolution, as stated in Brisson’s 2025 dynamic benchmark^6^. Existing methods attempt to address this problem by implementing temporal smoothing or continuity constraints to encourage consistency between adjacent snapshots^7^. Smoothing can lessen abrupt oscillations and increase short-term stability, but it also diminishes substantial structural transitions that occur naturally in dynamic networks. This is because smoothing suppresses the transitions that occur naturally. An excessive amount of smoothing can result in oversmoothing, which is a phenomenon in which the representations of nodes become excessively similar over time. This makes it more difficult for the model to identify true community reorganization. In light of this, the methodologies that are now in use are confronted with a basic trade-off between structural responsiveness and temporal stability^8^. Modelling approaches that place an emphasis on stability have a tendency to overlook significant community shifts, whereas models that place an emphasis on adaptation frequently experience community assignments that are unstable or noisy. This unresolved tension continues to be one of the most significant issues in dynamic community detection. This is especially true in streaming or poorly supervised environments, where reliable ground truth is not accessible and the model must continuously adapt to evolving graph topologies^9^.

We view spurious temporal fluctuations in this work as a basic learning failure in temporal graph neural networks. This failure is caused by the absence of explicit mechanisms that differentiate sustained structure evolution from high-frequency noise. In order to overcome this limitation, we propose a memory-guided stability regularization framework that allows for the explicit regularization of temporal representation learning through the utilization of historical structural references^10^. The system that has been presented does not enforce consistent smoothing across snapshots; rather, it keeps a structured memory of previous community configurations and uses it as an anchor to drive current representation modifications. This architecture enables the model to selectively suppress high-frequency spurious fluctuations while keeping low-frequency, community-consistent evolution. As a result, the model is able to stabilize temporal learning without compromising its adaptability to true structural changes^11^.

To be more specific, the approach that has been developed incorporates stability-aware constraints, which encourage node embeddings to continue to be consistent with previous structural patterns, unless sufficient evidence of an actual community transformation is found^12^. Through the incorporation of memory-guided regularization into the process of temporal propagation, the framework is able to effectively filter out changes that are caused by noise while yet preserving meaningful structural displacement. The presence of this mechanism lessens the likelihood of spurious temporal fluctuations and makes it possible to more accurately monitor the development of the community over extended periods of time^13^.

In order to test the proposed framework, we utilize a tight streaming environment. In this environment, graph snapshots are processed in a sequential manner without any access to future information^14^. This environment closely resembles dynamic situations that occur in the real world. The suggested method has been shown to consistently enhance community detection accuracy, temporal stability, and robustness against perturbations when compared with existing temporal GNN algorithms^1^. This has been demonstrated through extensive experiments conducted on both synthetic and real-world temporal networks. The findings demonstrate that the incorporation of memory-informed stability control provides an effective regularization strategy for addressing the trade-off between stability and responsiveness in dynamic community detection^15^. Additionally, it provides a reliable foundation for temporal graph learning in environments that are noisy and constantly changing.

The contribution of this work is positioned as a stability regularization extension to temporal graph neural networks, rather than as a new temporal learning paradigm. The main contributions are summarized as follows:

- We identify spurious temporal fluctuations as a practical source of instability in temporal GNN-based dynamic community detection, especially under noisy snapshot-level observations.
- We propose STR-GNN, a memory-guided stability regularization framework that anchors current node representations to historical structural summaries while allowing meaningful community evolution.
- We introduce an adaptive node-wise stability weighting mechanism that applies stronger regularization to nodes with large temporal displacement, thereby reducing unstable representation changes.
- We evaluate STR-GNN under a unified streaming protocol and provide additional ablation studies on memory, weighting, and stability regularization components.

## Related work

### Dynamic community detection

Early research on dynamic community detection extends traditional static clustering methods by incorporating temporal continuity constraints or evolutionary regularization to provide smooth transitions between consecutive graph snapshots. This was done in order to extend the capabilities of static clustering approaches. Methods such as evolutionary clustering, dynamic stochastic block models, and temporally regularized modularity optimization are examples of representative techniques. These methods make an effort to maintain historical community structures while allowing for incremental structural modifications over the course of time. Both the maintenance of temporal consistency and the prevention of abrupt partition changes brought on by noise or partial observations are successfully accomplished through the utilization of these strategies^16^.

However, the majority of conventional methods are dependent on global regularization tactics, which are designed to confine community assignments in a consistent manner across all nodes and time steps. The assumption that community evolution is smooth and slowly varying is frequently made by such global constraints. However, this assumption may not be valid in real-world dynamic networks, which are characterized by the coexistence of both genuine structural changes and false disturbances^17^. The result of this is that these models usually have difficulty striking a balance between the temporal stability and adaptability of their features. An excessive amount of regularization may prevent significant community transitions from occurring, whereas an inadequate amount of regularization might result in unstable or fragmented community assignments^18^. This is especially true when noisy temporal dynamics are present.

### Dynamic graph neural networks

A powerful framework for modeling dynamic graphs has been provided by recent developments in temporal graph neural networks (TGNNs). This framework allows for the learning of time-dependent node representations through recurrent updates, temporal attention mechanisms, or memory-based designs. Through the incorporation of structural information and temporal dependencies into unified embeddings, these models are able to enhance performance in a variety of downstream tasks, such as link prediction, node categorization, and dynamic community detection^19^.

Methods that are based on TGNN continue to be extremely sensitive to local perturbations in individual graph snapshots, despite the fact that they have a rather good representation capability. It is possible for modest structural alterations, missing edges, or brief interactions to be amplified during temporal aggregation^20^. This is because message passing propagates information between neighbors and over time. It is possible that the learned node embeddings will see significant fluctuations between consecutive snapshots as a result of this amplification effect. This is the case even when the underlying community structure remains to a large extent intact. As a consequence, the model might produce community assignments that are unstable or identify false community transitions that are not supported by the actual evolution of the graph, hence diminishing the dependability of dynamic community detection^21^. Recent studies have further advanced temporal graph learning and dynamic clustering from benchmark and multi-modal perspectives. Deep Temporal Graph Clustering provides a comprehensive benchmark and dataset suite for evaluating temporal graph clustering methods, emphasizing the importance of standardized datasets, consistent protocols, and fair comparison across dynamic graph models. Dictionary multi-modal temporal graph learning extends temporal graph learning to multi-modal settings by introducing dictionary-based temporal representation mechanisms^22,23^, showing that dynamic graph representations can benefit from structured temporal and cross-modal information^24,25^. These works highlight the need for stronger evaluation protocols and richer temporal representation mechanisms in dynamic graph learning^26,27^.

Compared with these studies, our work focuses specifically on temporal stability in dynamic community detection. Rather than introducing a new benchmark or multi-modal representation mechanism, STR-GNN introduces a memory-guided stability regularizer that explicitly reduces spurious temporal fluctuations in community assignments under noisy streaming conditions.

### Temporal smoothing and stability

In order to reduce the risk of instability in temporal representation learning, a number of research have proposed the implementation of temporal smoothing, exponential averaging, or continuity regularization. Existing methods attempt to address this problem by implementing temporal smoothing or continuity constraints to encourage consistency between adjacent snapshots^28^. These strategies confine node representations to change gradually over time, which results in a reduction in high-frequency fluctuations and an improvement in short-term stability during the process. In order to prevent abrupt embedding shifts brought on by noise or inadequate observations, such smoothing-based techniques have been widely employed in the field of temporal graph learning^29^.

Uniform smoothing, on the other hand, applies the same level of temporal constraint to each and every node and each and every time step, regardless of whether the change that is detected is the result of noise or true structural evolution. Excessive smoothing can suppress significant transitions and lead to oversmoothing in dynamic networks, which is when node representations become extremely similar over time. This can occur when communities split, merge, or rearrange themselves. Due to this, the model’s capacity to accurately capture genuine structural drift is diminished, and its responsiveness to actual community changes is diminished as well. On the other hand, decreasing the smoothing strength may bring back adaptability, but it frequently brings back instability. This suggests that the approaches that are now in use do not have mechanisms that can differentiate between spurious temporal variations and actual structural updates inside the system. As a consequence, stability is often considered to be a side effect of smoothing rather than a selective and structure-aware objective in the context of temporal graph learning^30^.

### Perspective

We explicitly interpret unstable community evolution in temporal graph neural networks as a manifestation of spurious temporal fluctuations, in which the model produces artificial structural changes due to sensitivity to high-frequency perturbations rather than genuine graph evolution. This perspective highlights the fact that instability in dynamic community recognition is not just a result of insufficient smoothing, but also a result of the absence of mechanisms that can selectively retain relevant temporal information while filtering out noise-induced changes. This is because these mechanisms are unable to selectively preserve significant temporal information^31^.

For the purpose of overcoming this limitation, we present a framework for memory-guided stability regularization that integrates historical structural references into the process of temporal learning. The technique that has been proposed does not make use of uniform smoothing; rather, it keeps a structured memory of previous community configurations and makes use of this memory to guide the modifications that are being made to the representation. By utilizing this architecture, the model is able to minimize spurious fluctuations that occur at high frequencies while also preserving low-frequency dynamics that are compatible with the community and reflect the actual evolution of the structure. The suggested perspective extends the temporal graph models that are already in existence and offers a systematic approach to resolve the long-standing trade-off between adaptability and stability in dynamic community detection^32^. This is accomplished by making stability a selective and structure-aware objective.

In streaming or partially observed dynamic networks, where models are required to function without access to future information, this viewpoint is especially pertinent. As a result, these networks are more susceptible to noise-induced instability. An important gap in the current research is filled by the proposed framework, which offers a more reliable foundation for community detection in continuously evolving graphs^33^. This is accomplished by integrating memory-informed stability constraints into temporal representation learning.

## Methodology

The core idea of STR-GNN is to introduce stability control into temporal graph representation learning without replacing the underlying temporal GNN encoder. In dynamic community detection, the current node representations should not change abruptly unless the current graph snapshot provides sufficient evidence of genuine community evolution. However, standard temporal GNNs may overreact to short-lived perturbations, missing edges, noisy interactions, or incomplete observations. Such overreaction can cause unstable community assignments, including artificial splits, merges, or oscillatory membership changes across adjacent snapshots.

To address this issue, STR-GNN keeps current node representations close to historical structural summaries through a memory-guided stability regularization mechanism. The structural memory stores compact summaries of previous graph states and provides a temporal reference for evaluating whether a new representation update is consistent with recent community evolution. Instead of enforcing uniform smoothing across all nodes and all time steps, the proposed method selectively penalizes unstable deviations that are large, abrupt, and weakly supported by historical structure.

This design allows STR-GNN to preserve meaningful community transitions while suppressing spurious temporal fluctuations caused by transient noise. Therefore, the method should be understood as a stability-regularized extension to temporal GNNs rather than a completely new temporal architecture. The following sections formalize the dynamic graph setting, community projection, structural memory, stability regularization, online update procedure, concrete implementation details, and scalability properties of the proposed framework.

### Method overview

**Fig. 1:**
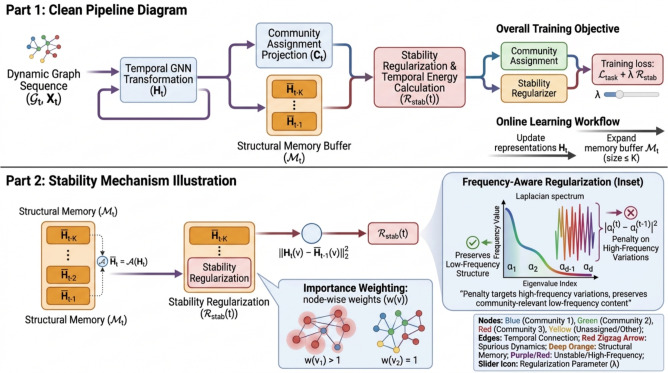
Revised overview of STR-GNN. Part 1 shows the clean temporal learning pipeline, including dynamic graph input, temporal GNN encoding, community projection, structural memory, stability regularization, and the final objective. Part 2 illustrates the memory-guided stability mechanism, where historical structural summaries and node-wise weights penalize unstable temporal deviations. The frequency-aware inset shows that the regularizer suppresses high-frequency spurious fluctuations while preserving low-frequency community-relevant structure.

Figure 1 provides the revised visual overview of STR-GNN. In response to the concern that the original figure was overly complex and difficult to follow, we reorganize the illustration into two parts. The first part presents the overall temporal learning pipeline, including dynamic graph input, temporal GNN transformation, community assignment projection, structural memory buffer, stability regularization, and the final training objective. The second part focuses specifically on the stability mechanism, showing how memory anchors, node-wise importance weights, and frequency-aware regularization jointly suppress spurious temporal fluctuations.

### Problem setting and temporal dynamics

We consider a dynamic graph as an ordered sequence of graph snapshots

1 $$\begin{aligned} \mathscr {G} = \{ G_t = (V_t, E_t, X_t) \}_{t=1}^{T}, \end{aligned}$$

where $V_t$ and $E_t$ denote the node and edge sets at time step *t*, respectively, and $X_t \in \mathbb {R}^{|V_t| \times d}$ denotes the matrix of node features. Depending on the application scenario, the node set may remain fixed across time or may change dynamically as nodes appear or disappear. The objective of dynamic community detection is to recover a temporally consistent sequence of community assignments

2 $$\begin{aligned} \mathscr {C} = \{ C_t \}_{t=1}^{T}, \end{aligned}$$

where $C_t$ describes the community structure of snapshot $G_t$.

Compared with static community detection, the temporal setting introduces two additional sources of difficulty. First, graph topology evolves over time due to the emergence, disappearance, or reweighting of interactions. Second, the observed changes are not always informative. In real-world dynamic graphs, short-term perturbations may originate from sampling incompleteness, measurement errors, transient interactions, non-stationary noise, or abrupt but semantically unimportant events. These perturbations may not reflect a genuine reorganization of community structure, but they can still significantly affect temporal representation learning. As a result, a dynamic community detector must not only identify meaningful structural evolution, but also discriminate between persistent community changes and short-lived fluctuations.

Let $H_t \in \mathbb {R}^{|V_t| \times k}$ denote the latent node representations at time *t*. A broad class of temporal graph neural networks can be abstracted as a nonlinear temporal operator

3 $$\begin{aligned} H_t = \mathscr {T}_\theta (H_{t-1}, G_t), \end{aligned}$$

where $\mathscr {T}_\theta (\cdot )$ integrates spatial message passing on the current graph snapshot with temporal propagation from previous representations. This abstraction covers recurrent temporal GNNs, attention-based temporal architectures, memory-augmented graph models, and hybrid temporal propagation schemes.

Although such models provide strong expressive power for dynamic graph representation learning, they may become excessively sensitive to local perturbations in $G_t$ or $X_t$. When the temporal operator reacts strongly to short-term noise, the learned representations may exhibit abrupt changes even when the underlying community structure remains largely stable. These unstable representation shifts can accumulate across time and produce illusory community transitions, such as artificial splits, merges, or oscillatory membership changes. We refer to this phenomenon as *spurious temporal dynamics*, namely, temporal variation in latent representations that is not supported by true structural evolution. In the context of dynamic community detection, such dynamics manifest as spurious temporal fluctuations in community assignments and reduce both the stability and reliability of temporal inference.

To address this issue, our formulation treats temporal stability as a selective structural objective rather than a uniform smoothing heuristic. Instead of merely enforcing similarity between adjacent snapshots, we aim to stabilize representation evolution relative to historical structural evidence. This perspective motivates the introduction of a memory-guided temporal reference mechanism, which allows the model to regulate unstable updates without suppressing meaningful long-range community transitions.

### Community assignment as latent projection

Given the latent node representations $H_t$, we model community inference as a projection from representation space into a latent community space:

4 $$\begin{aligned} C_t = \Pi _{\mathscr {C}}(H_t), \end{aligned}$$

where $\Pi _{\mathscr {C}}(\cdot )$ denotes a differentiable projection operator producing either soft community memberships or hard assignments. This projection may be implemented by a clustering head, a prototype-based assignment module, a learned partitioning operator, or other differentiable community decoding mechanisms.

This formulation provides a convenient interface between representation learning and community inference. Temporal GNNs first encode the evolving graph into node embeddings, and the community projection then transforms these embeddings into community structures. Under this view, community detection is not treated as a separate post-processing step, but as a downstream structural interpretation of the latent dynamics learned by the model.

However, this coupling also reveals an important vulnerability. Because community assignments are directly induced from $H_t$, instability in the latent representation space propagates immediately into the community space. Even relatively small perturbations in node embeddings may move a node across cluster boundaries, especially in settings where inter-community separation is weak or the graph is only partially observed. Consequently, temporal inconsistency in $H_t$ may lead to abrupt and structurally implausible changes in $C_t$, even if the underlying graph has not undergone a meaningful reorganization.

This observation is central to our method. Rather than regulating the community assignments only after they have been produced, we focus on stabilizing the representation dynamics that generate them. If the temporal evolution of $H_t$ can be constrained to remain structurally coherent, then the resulting community assignments become more reliable, more interpretable, and less vulnerable to spurious temporal fluctuations. Accordingly, the proposed framework introduces explicit stability control at the representation level before the projection step, allowing the community projection $\Pi _{\mathscr {C}}(\cdot )$ to operate on temporally grounded and less noisy embeddings.

### Structural memory as a temporal reference

To regulate temporal instability, we introduce a structural memory that stores historical representations as reference states. The purpose of this memory is not to recursively propagate hidden states in the manner of recurrent networks, but rather to maintain a historical reference manifold that summarizes persistent structural information across past snapshots. This memory provides an external anchor against which current representations can be compared, thereby helping the model distinguish transient fluctuations from meaningful structural evolution.

Given the node embeddings $H_t$, we first compute an aggregated structural summary

5 $$\begin{aligned} \bar{H}_t = \mathscr {A}(H_t), \end{aligned}$$

where $\mathscr {A}(\cdot )$ is a permutation-invariant aggregation operator. Depending on the implementation, $\mathscr {A}(\cdot )$ may correspond to mean pooling, attention-weighted summarization, prototype extraction, or community-aware structural compression. The role of this aggregation is to transform the snapshot-level embedding space into a compact structural descriptor that preserves the dominant patterns of the current graph state while filtering out node-level redundancy.

The memory buffer at time *t* is then defined as

6 $$\begin{aligned} \mathscr {M}_t = \{ \bar{H}_{t-\tau } \mid \tau = 1,\dots ,K \}, \end{aligned}$$

where *K* denotes the memory horizon. This buffer stores the most recent *K* structural summaries and therefore encodes short-to-medium-term temporal context. Unlike standard recurrent hidden states, which are directly transformed at each step and may accumulate model bias, the memory entries are stored as historical reference snapshots. This design reduces the risk of error amplification and allows the current representation to be evaluated against a more stable temporal backdrop.

The key intuition is that meaningful community evolution typically exhibits a degree of structural continuity over time, whereas spurious fluctuations tend to be short-lived and weakly supported by historical patterns. By consulting a memory of past structural states, the model can assess whether a new representation update is consistent with long-term graph evolution or merely reflects a temporary perturbation. As a result, structural memory acts as a temporal reference system that grounds current learning in historical evidence.

Another advantage of this formulation is that it separates temporal stabilization from explicit recurrent dependence. The memory does not force the model to strictly preserve past representations, nor does it impose uniform temporal smoothing. Instead, it provides a flexible set of structural references that can be used to guide current updates. This enables the model to remain responsive to genuine structural changes while suppressing unstable variations unsupported by temporal history.

In the proposed framework, structural memory therefore plays three roles: it encodes persistent community-related structure, provides a historical anchor for stability evaluation, and offers an interpretable reference mechanism for distinguishing authentic evolution from spurious temporal fluctuations.

### Difference from memory-based temporal GNNs

Although STR-GNN uses a memory component, its role differs from the memory module in models such as TGN. In TGN, memory is primarily used as a node-level hidden state for temporal information propagation and event history encoding. It helps update node representations as new interactions arrive. In contrast, the memory in STR-GNN is not used as the main temporal encoder. Instead, it serves as a structural reference for stability regularization.

Specifically, STR-GNN stores compact historical structural summaries and uses them to penalize unstable deviations in current representations. The goal is not merely to remember past interactions, but to distinguish persistent structural evolution from short-lived perturbations. Therefore, the proposed memory module acts as a regularization anchor, whereas TGN-style memory acts as a representation propagation mechanism.

This distinction is important for dynamic community detection because stable community tracking requires not only temporal information propagation, but also explicit control of spurious temporal fluctuations. STR-GNN can therefore be applied on top of temporal encoders such as TGAT or TGN, while its memory-guided regularization remains a separate stability-control mechanism.

### Stability regularization via temporal energy

To quantify the degree of spurious temporal dynamics, we define a temporal deviation energy that measures how far current node representations deviate from memory-induced historical references:

7 $$\begin{aligned} \mathscr {E}_t(H_t) = \mathbb {E}_{v \in V_t} \Big [ \big \Vert H_t(v) - \mathscr {P}_{\mathscr {M}_t}(H_t(v)) \big \Vert _2^2 \Big ], \end{aligned}$$

where $\mathscr {P}_{\mathscr {M}_t}(\cdot )$ denotes projection onto the memory-induced reference subspace. Intuitively, this quantity measures the amount of temporal energy that cannot be explained by previously observed structural patterns. A larger deviation energy indicates that the current representation update moves further away from historically consistent configurations, which may signal instability or spurious temporal fluctuations.

In the most general setting, the operator $\mathscr {P}_{\mathscr {M}_t}(\cdot )$ projects node representations onto a reference subspace spanned by memory entries. This interpretation allows the regularizer to be viewed as a temporal consistency constraint relative to a learned historical manifold rather than a simple pairwise penalty between adjacent snapshots. However, to keep the formulation computationally practical in online settings, we approximate this projection using the most recent memory state:

8 $$\begin{aligned} \mathscr {P}_{\mathscr {M}_t}(H_t(v)) \approx \bar{H}_{t-1}(v). \end{aligned}$$

This approximation preserves the essential idea of memory-grounded temporal reference while avoiding the overhead of more expensive subspace projection operations.

A crucial limitation of uniform temporal regularization is that it assumes all deviations should be penalized equally. In dynamic community detection, this assumption is overly restrictive, because some nodes may genuinely change their community behavior while others only fluctuate due to noise. To account for this heterogeneity, we introduce a node-wise importance weight

9 $$\begin{aligned} w(v) = \sigma \!\left( \Vert H_t(v) - H_{t-1}(v) \Vert _2 \right) , \end{aligned}$$

where $\sigma (\cdot )$ is a monotonic scaling function. This weight acts as an adaptive indicator of temporal instability. Nodes that undergo larger representation changes receive stronger stabilization pressure, whereas nodes with already stable trajectories are regularized more lightly.

Using this weighting mechanism, the final stability regularizer is defined as

10 $$\begin{aligned} \mathscr {R}_{\textrm{stab}}(t) = \mathbb {E}_{v \in V_t} \left[ w(v) \cdot \big \Vert H_t(v) - \bar{H}_{t-1}(v) \big \Vert _2^2 \right] . \end{aligned}$$

This regularizer differs fundamentally from ordinary smoothing penalties. Rather than globally shrinking all temporal differences, it selectively penalizes unstable deviations relative to a structural memory anchor. The effect is to suppress representation changes that are large, temporally abrupt, and poorly supported by recent structural history, while still permitting substantial changes when they are consistent with evolving community organization.

From an optimization perspective, this formulation also provides a natural bridge between stability and adaptability. If *λ* is too small, the model behaves like a standard temporal GNN and remains highly responsive but unstable. If *λ* is too large, the model becomes overly conservative and may underfit genuine transitions. The weighted temporal energy term therefore plays a balancing role: it discourages ungrounded high-magnitude deviations while preserving the possibility of meaningful structural reconfiguration.

In this sense, the proposed regularizer can be interpreted as an anti-spurious mechanism at the representation level. It does not simply reduce variance; it constrains temporal learning to remain structurally plausible relative to historical evidence. This makes the resulting community assignments more temporally coherent, less oscillatory, and more robust in noisy streaming environments.

### Frequency-aware interpretation

The proposed stability framework can also be interpreted from the viewpoint of graph signal processing. For each snapshot, the node representations may be expanded in the eigenbasis of the graph Laplacian:

11 $$\begin{aligned} H_t = \sum _{i=1}^{|V_t|} \alpha _i^{(t)} \, \phi _i^{(t)}, \end{aligned}$$

where $\phi _i^{(t)}$ are eigenvectors of the graph Laplacian $\textbf{L}_t$, and $\alpha _i^{(t)}$ are the corresponding spectral coefficients. Under this decomposition, different spectral components capture different modes of variation across the graph.

Low-frequency components correspond to smooth structural patterns over the graph and are commonly associated with large-scale community organization. These components vary gradually across neighboring nodes and often encode persistent mesoscopic structures such as communities, blocks, or stable interaction regimes. By contrast, high-frequency components correspond to rapid local variation, which is more sensitive to isolated perturbations, noisy edge changes, or local inconsistencies. In dynamic graphs, such high-frequency components are especially vulnerable to transient snapshot noise and may therefore dominate unstable short-term representation fluctuations.

The proposed stability regularization implicitly suppresses the temporal energy associated with these unstable spectral modes. In particular, it discourages rapid changes in the spectral coefficients of high-frequency components:

12 $$\begin{aligned} \sum _{i \in \mathscr {F}_{\textrm{high}}} \big | \alpha _i^{(t)} - \alpha _i^{(t-1)} \big |^2, \end{aligned}$$

while preserving the lower-frequency components that are more likely to reflect consistent community evolution. This perspective clarifies why the proposed approach differs from naive temporal smoothing. Conventional smoothing often shrinks all temporal variation uniformly, including those low-frequency changes that correspond to meaningful community transitions. In contrast, the memory-guided stability mechanism acts more selectively: it attenuates unstable, high-frequency fluctuations while retaining smoother and structurally consistent evolution.

This frequency-aware interpretation is important because it connects the empirical behavior of the model to a principled notion of signal quality on dynamic graphs. Spurious temporal dynamics can be viewed as a form of unstable high-frequency temporal energy that contaminates the evolving node representations. The proposed regularization reduces this contamination and therefore improves the structural fidelity of the latent dynamics. As a result, the learned representations remain sensitive to genuine community drift but are less likely to overreact to noisy local perturbations.

### Overall objective

The learning objective over the temporal horizon is defined as

13 $$\begin{aligned} \min _\theta \sum _{t=1}^{T} \Big ( \mathscr {L}_{\textrm{task}}(C_t) + \lambda \, \mathscr {R}_{\textrm{stab}}(t) \Big ), \end{aligned}$$

where $\mathscr {L}_{\textrm{task}}(C_t)$ denotes the community detection loss associated with snapshot *t*, and *λ* controls the trade-off between temporal adaptability and stability regularization.

The task loss can be instantiated according to the supervision regime. In fully supervised settings, it may correspond to a classification or cross-entropy objective over community labels. In weakly supervised or unsupervised settings, it may be formulated through clustering consistency, modularity-oriented objectives, contrastive structure learning, or prototype-based assignment losses. This flexibility allows the proposed framework to be combined with a broad range of community inference strategies without depending on a single supervision paradigm.

The second component, $\mathscr {R}_{\textrm{stab}}(t)$, plays the role of a temporal structural prior. Instead of forcing strict equality between adjacent embeddings, it guides the optimization toward representations that remain close to historically grounded structural states unless the data provide sufficient evidence of meaningful change. The coefficient *λ* therefore determines how strongly the model is encouraged to preserve temporal coherence. A larger *λ* favors stronger stabilization and greater resistance to noise, whereas a smaller *λ* increases flexibility and responsiveness to sudden structural reorganization.

Importantly, the objective is not intended to eliminate temporal variation. Dynamic community detection requires the model to remain responsive to community splits, merges, births, disappearances, and reassignments. The purpose of the stability term is only to penalize those representation changes that are disproportionately large relative to the available historical context. In this way, the full objective balances two competing goals: structural responsiveness to genuine evolution and resistance to noisy, spurious temporal fluctuations.

From an optimization standpoint, this formulation may also be viewed as a regularized temporal risk minimization problem. The task loss encourages the model to explain the current snapshot, while the stability regularizer constrains that explanation to remain compatible with historical structure. This combination yields a more reliable temporal learning process, particularly in online settings where future snapshots are unavailable and the model must make decisions incrementally.

### Online learning dynamics

A key requirement of dynamic community detection is the ability to operate in streaming environments, where graph snapshots arrive sequentially and the model cannot access future information. To accommodate this setting, the proposed framework is designed as an online learning procedure in which both latent representations and structural memory are updated incrementally over time.

At each time step, the online update takes the form

14 $$\begin{aligned} (H_t, \mathscr {M}_t) \leftarrow (\mathscr {T}_\theta (H_{t-1}, G_t), \mathscr {M}_{t-1} \cup \{\bar{H}_t\}), \end{aligned}$$

with the memory size constrained to *K*. This formulation means that current inference depends only on previously computed representations, the current graph snapshot, and the stored memory buffer. No future graph states are required at any stage of the update.

This incremental structure provides several practical advantages. First, it makes the method suitable for real-time or near-real-time dynamic graph analysis, where graph observations are continuously received. Second, it reduces temporal information leakage, since the model is never allowed to condition on future snapshots during training or inference. Third, it supports evaluation under strict streaming protocols, which more faithfully reflect deployment conditions than transductive or bidirectional temporal settings.

The role of memory in this online formulation is particularly important. Because the model cannot observe future graph structure, it must assess the significance of current temporal deviations using only historical evidence. The memory buffer therefore serves as a compact historical context that allows the model to judge whether a newly observed change is structurally plausible. This mechanism is especially valuable in noisy streaming environments, where abrupt but short-lived perturbations can otherwise trigger unstable representation updates.

By constraining memory size to *K*, the framework also maintains bounded storage and computational overhead. This makes the method scalable to long temporal horizons without requiring unbounded accumulation of past states. The resulting online learning dynamics therefore provide a practical and effective regularization strategy for temporally stable community detection in evolving graphs.

### Theoretical perspective on stability-constrained updates

The proposed stability mechanism can be further interpreted through the lens of regularized temporal optimization. Consider the unregularized temporal update

15 $$\begin{aligned} H_t = \mathscr {T}_\theta (H_{t-1}, G_t), \end{aligned}$$

which may respond strongly to perturbations in the current graph snapshot. To understand the effect of stability regularization, we consider an implicit proximal form of the update in which the representation at time *t* is encouraged to remain close both to the temporal GNN prediction and to the historical reference state:

16 $$\begin{aligned} H_t^{*} = \arg \min _{H} \left( \Vert H-\mathscr {T}_\theta (H_{t-1},G_t)\Vert _2^2 + \lambda \Vert H-\bar{H}_{t-1}\Vert _2^2 \right) . \end{aligned}$$

The minimizer of this quadratic objective is

17 $$\begin{aligned} H_t^{*} = \frac{ \mathscr {T}_\theta (H_{t-1},G_t) + \lambda \bar{H}_{t-1} }{1+\lambda }. \end{aligned}$$

This expression shows that the proposed method can be viewed as a stability-constrained interpolation between the raw temporal GNN update and the memory-based historical anchor. When *λ =0*, the update reduces to the original temporal GNN. As *λ* increases, the update becomes increasingly grounded in the historical structural reference.

This interpretation clarifies why the method suppresses spurious temporal fluctuations. If a current representation update deviates sharply from recent structural history without sufficient support from persistent graph evolution, the memory-guided term pulls the representation back toward a more plausible state. Conversely, when structural change is strong and consistent over time, the task-driven temporal update can still dominate the optimization. Thus, the proposed framework is not merely conservative; it is selectively stabilizing.

We may also characterize the effect of the regularizer on temporal deviation. Let

18 $$\begin{aligned} \Delta _t = \Vert H_t-H_{t-1}\Vert . \end{aligned}$$

If the temporal operator is Lipschitz continuous with respect to both representations and graph perturbations, then in the absence of stability control the deviation may satisfy a relation of the form

19 $$\begin{aligned} \Delta _t \le L_H \Delta _{t-1} + L_G \Vert G_t-G_{t-1}\Vert , \end{aligned}$$

where $L_H$ and $L_G$ are sensitivity coefficients. Under the stability-constrained formulation, the effective deviation is reduced to

20 $$\begin{aligned} \Delta _t \le \frac{L_H}{1+\lambda }\Delta _{t-1} + \frac{L_G}{1+\lambda }\Vert G_t-G_{t-1}\Vert . \end{aligned}$$

Hence, the amplification of temporal perturbations is attenuated by the factor $(1+\lambda )^{-1}$. This result supports the intuition that stability regularization reduces the propagation of noisy temporal fluctuations across time.

From a robustness viewpoint, suppose the current graph snapshot is perturbed as

21 $$\begin{aligned} G_t' = G_t + \epsilon _t. \end{aligned}$$

Without memory-guided stability control, the corresponding embedding error may scale proportionally with $\Vert \epsilon _t\Vert$. With the proposed regularizer, the impact of this perturbation is damped by the same stabilizing factor, indicating that the representation trajectory becomes less sensitive to snapshot-level noise. This theoretical perspective aligns with the practical goal of reducing artificial community transitions in noisy temporal graphs.

### Algorithmic procedure

**Algorithm 1:**
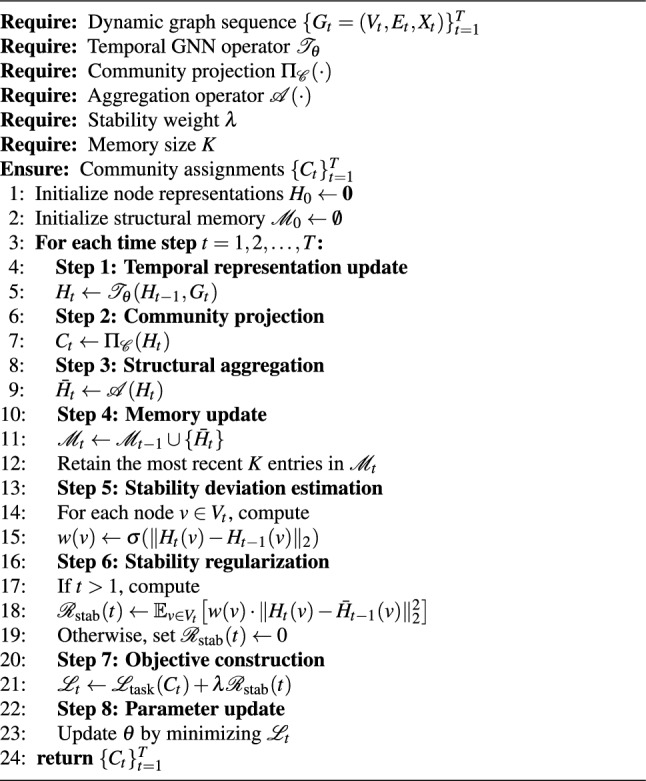
Stability-aware temporal GNN for dynamic community detection

Algorithm 1 summarizes the complete temporal learning procedure. At each time step, the temporal GNN first updates node embeddings from the current graph snapshot and the previous latent state. These embeddings are then projected into a latent community space to obtain the current community assignments. Next, an aggregated structural summary is computed and inserted into the memory buffer, which provides historical structural context for future time steps.

When *t>1*, the method evaluates the magnitude of node-wise temporal change and constructs adaptive importance weights that emphasize unstable nodes. Using these weights, the stability regularizer penalizes deviations between current embeddings and the historical memory reference. The model parameters are then updated by jointly minimizing the community detection loss and the temporal stability term.

This procedure highlights the central design principle of the method: dynamic community detection is treated as a sequential inference problem in which current predictions must remain compatible with temporally grounded structural evidence. Because all computations depend only on current and past information, the algorithm is naturally compatible with online deployment and strict streaming evaluation.

### Concrete model instantiation and implementation details

To address reproducibility concerns, we instantiate the proposed STR-GNN framework as a concrete temporal graph learning model rather than leaving its components abstract. In all experiments, the temporal representation operator $\mathscr {T}_{\theta }$ is implemented using a TGAT-style temporal attention encoder. For each snapshot $G_t=(V_t,E_t,X_t)$, the encoder performs graph-based message aggregation over the current topology and applies temporal attention over historical node representations from previous snapshots. The output is the node embedding matrix $H_t \in \mathbb {R}^{|V_t|\times d}$, where the embedding dimension is fixed to *d=128* for all neural models.

The community projection operator $\Pi _{\mathscr {C}}(\cdot )$ is implemented as a clustering-based assignment module. After obtaining $H_t$, we apply *k*-means clustering to the node embeddings at each time step to obtain community assignments $C_t$. When ground-truth labels are available, the number of clusters is set to the number of reference communities. For datasets with partial or unavailable labels, the number of clusters is selected according to the best validation modularity on the training snapshots and then fixed for all test snapshots.

The aggregation operator $\mathscr {A}(\cdot )$ is implemented by mean pooling:

22 $$\begin{aligned} \bar{h}_t = \frac{1}{|V_t|} \sum _{v\in V_t} H_t(v), \end{aligned}$$

where $\bar{h}_t \in \mathbb {R}^{d}$ is the snapshot-level structural summary.

The memory buffer stores the most recent $K_m=3$ structural summaries:

23 $$\begin{aligned} M_t=\{\bar{h}_{t-1},\bar{h}_{t-2},\ldots ,\bar{h}_{t-K_m}\}. \end{aligned}$$

The stability weight function is instantiated as:

24 $$\begin{aligned} w_t(v)= \sigma \left( \left\| H_t(v)-H_{t-1}(v)\right\| _2 \right) , \end{aligned}$$

where $\sigma (\cdot )$ denotes the sigmoid function. This design assigns larger weights to nodes whose embeddings change sharply between consecutive snapshots, so unstable nodes receive stronger regularization pressure.

The implemented stability regularizer is:

25 $$\begin{aligned} { R_{\textrm{stab}}(t)= \frac{1}{|V_t|} \sum _{v\in V_t} w_t(v) \left\| H_t(v)-\bar{h}_{t-1} \right\| _2^2. } \end{aligned}$$

Here, $\bar{h}_{t-1}$ is the most recent structural memory anchor. This implementation follows the memory-guided approximation described above and avoids expensive subspace projection during streaming inference.

The full training objective is:

26 $$\begin{aligned} { \mathscr {L}_t = \mathscr {L}_{\textrm{task}}(t) + \lambda R_{\textrm{stab}}(t). } \end{aligned}$$

For datasets with ground-truth community labels, $\mathscr {L}_{\textrm{task}}$ is implemented as cross-entropy loss over community labels using a linear classification head attached to $H_t$. For datasets without full labels, $\mathscr {L}_{\textrm{task}}$ is implemented as an unsupervised modularity-oriented objective. All neural models are trained using the Adam optimizer with learning rate $10^{-3}$, weight decay $5\times 10^{-4}$, dropout rate 0.3, batch size 1 snapshot, and a maximum of 200 epochs. Early stopping is applied if the validation loss does not improve for 20 consecutive epochs.

For fair comparison, TGAT, DySAT, EvolveGCN, TGN, and STR-GNN use the same embedding dimension, optimizer, learning rate, dropout rate, and stopping criterion. Model-specific hyperparameters for baselines are selected following their original recommended settings and then tuned on the validation snapshots. All results are reported over three independent runs with different random seeds.

### Scalability discussion

The proposed method is implemented on static graph snapshots, which means that each temporal graph is processed as a sequence of adjacency-based graph instances. This design has clear advantages and limitations for large-scale dynamic graphs. The main advantage is that snapshot-based processing is simple, modular, and compatible with many existing temporal GNN backbones and community detection baselines. It also allows the model to operate in a strict streaming setting, where only the current snapshot and historical memory are available.

The computational cost of one snapshot update is mainly determined by message passing over the current graph and the stability regularization term. If sparse adjacency representation is used, the message-passing cost is approximately $O(|E_t|d)$, where $|E_t|$ is the number of edges at time *t* and *d* is the embedding dimension. The stability regularizer introduces an additional node-wise cost of $O(|V_t|d)$, because it compares current node embeddings with the memory anchor. Therefore, the total per-snapshot complexity can be written as:

27 $$\begin{aligned} { O(|E_t|d + |V_t|d). } \end{aligned}$$

The memory overhead is bounded because the model only stores the most recent $K_m$ structural summaries rather than the full historical graph sequence. With mean-pooled structural memory, the memory cost is $O(K_m d)$. If node-level memory is used, the cost becomes $O(K_m |V_t| d)$, which is more expensive and should be avoided for very large graphs.

The main limitation is that snapshot construction may become costly when graphs are extremely large, dense, or have very high-frequency edge updates. In such cases, repeatedly materializing adjacency matrices may increase memory usage and preprocessing time. The proposed method is therefore most suitable for sparse large-scale dynamic graphs processed with sparse adjacency matrices or mini-batch neighbor sampling. For extremely dense or event-level temporal graphs, future work should extend the framework to continuous-time temporal graph learning without explicitly constructing full snapshots.

## Experiments

This section evaluates the proposed memory-guided stability framework for dynamic community detection. The experiments examine three aspects: (i) alignment with reference communities, (ii) temporal consistency under evolving graph dynamics, and (iii) robustness against noise-induced temporal perturbations. All methods are evaluated under a standardized online protocol to ensure fair temporal comparison.

### Datasets

We use a unified benchmark suite throughout all experiments. To avoid inconsistency across tables, all main results, robustness tests, ablation studies, and statistical analyses are conducted on the same six datasets: Cora-Temporal, Citeseer-Temporal, DBLP-Dynamic, Enron-Email, Reddit-Interaction, and SBM-Drift. These datasets cover citation, collaboration, communication, social interaction, and synthetic dynamic graph scenarios (Table 1).

**Table 1:** Unified dynamic graph benchmark suite used in all experiments.

Dataset	Domain	Snapshots	Label type	Setting
Cora-temporal	Citation	10	Full	Supervised
Citeseer-temporal	Citation	10	Full	Supervised
DBLP-dynamic	Coauthor	15	Partial	Weakly supervised
Enron-email	Communication	12	Partial	Weakly supervised
Reddit-interaction	Social	12	None	Unsupervised
SBM-drift	Synthetic	20	Full	Supervised

### Learning setting and label usage

The learning setting is defined separately for each dataset according to label availability. For cora-temporal, citeseer-temporal, and SBM-drift, full ground-truth community labels are available; therefore, supervised training uses cross-entropy loss and evaluation reports NMI and ARI against reference labels. For DBLP-dynamic and enron-email, only partial labels are available; therefore, labeled nodes are used for weak supervision, while all nodes are included during community assignment and evaluation. For Reddit-Interaction, no ground-truth community labels are available; therefore, the model is trained using the modularity-oriented unsupervised objective and evaluated using modularity *Q* and temporal variation.

All compared methods are evaluated under the same label-availability condition for each dataset. No method is allowed to use labels that are unavailable to other baselines. In the streaming setting, at time step *t*, models only access $G_t$, historical representations, and previously stored memory. Future snapshots are never used during training or inference.

### Baselines

We compare against representative baselines spanning snapshot-based, probabilistic, and neural temporal models:

- *Louvain/Leiden/Infomap* static community detection executed independently for each snapshot;
- *FacetNet, dynamic SBM, evolutionary clustering (EC)* probabilistic temporal models incorporating explicit smoothness assumptions;
- *EvolveGCN, TGAT, DySAT, TGN* temporal graph neural networks that model dynamic node representations;

We discuss these recent methods and include them in the extended comparison where comparable implementations or reproducible settings are available. To strengthen the comparison, we additionally include recent or stability-oriented baselines, including deep temporal graph clustering (DTGC), dictionary multi-modal temporal graph learning (DMTGL), and Bayesian GNN smoothing. These methods provide stronger reference points beyond standard temporal GNNs such as TGAT, DySAT, EvolveGCN, and TGN.

### Experimental protocol and reproducibility

All methods are evaluated under a strictly streaming setting that preserves temporal causality. At each time step *t*, models process only the current snapshot $G_t$ together with representations from time $t\!-\!1$, without access to future information. The proposed framework maintains a compact structural memory of size *K=3*, storing aggregated representations from recent snapshots and updating them in a first-in-first-out manner to ensure online consistency.

To assess robustness against spurious temporal fluctuations, we inject controlled perturbations at each snapshot, including random edge flips (5% and 10%) and additive Gaussian noise on node features (*σ =0.1*), while preserving the underlying community structure. Performance is evaluated using NMI and ARI when ground-truth labels are available, temporal variation (Temp-Var) to measure stability, and modularity *Q* to assess structural cohesion. All metrics are computed per snapshot and averaged over time. Experiments are repeated three times with fixed random seeds to ensure reproducibility. Temporal snapshots are constructed by sorting all timestamped edges chronologically and dividing them into equal-width temporal windows. For citation and coauthor datasets, snapshots are constructed according to publication year or time interval. For communication and interaction datasets, snapshots are constructed according to interaction timestamps. Isolated nodes are retained to preserve node-set consistency across time.

Node features are prepared as follows. For citation datasets, bag-of-words node features are used when available. For datasets without original node attributes, we use degree-based structural features combined with one-hot node identity features projected into a low-dimensional embedding space. All features are normalized before training.

Baseline hyperparameters are tuned on validation snapshots using the search ranges recommended in the original papers. For fair comparison, all neural baselines use the same embedding dimension, optimizer, learning rate, dropout rate, maximum epoch number, and early-stopping patience. The best validation configuration is fixed before test evaluation.

All experiments are repeated over three independent runs with different random seeds. We report mean ± standard deviation for all main results. Statistical significance is assessed using paired two-tailed *t*-tests between STR-GNN and the strongest baseline on each dataset. Differences with *p<0.05* are considered statistically significant.

### Aggregate results

Tables 2 and 3 present the main comparison on the unified benchmark suite. The results are evaluated across NMI, ARI, modularity *Q*, and temporal variation (TV). STR-GNN achieves the highest NMI and ARI on datasets with available reference or partial community labels, while temporal smooth-CD obtains lower TV and higher modularity in several cases. This pattern indicates that STR-GNN improves community alignment while maintaining competitive temporal stability, whereas strong smoothing methods favor conservative partitions. Results are assessed across four complementary dimensions: community alignment (NMI, ARI), structural cohesiveness (modularity *Q*), and temporal consistency (Temp-Var).

Snapshot-based community detection approaches demonstrate significant temporal volatility across all datasets. Each snapshot is processed independently, leading to minor perturbations or sampling noise that frequently cause sudden community shifts, resulting in elevated Temp-Var values and erratic temporal behavior.

Probabilistic temporal models and post-hoc smoothing techniques mitigate temporal variation by imposing explicit smoothness restrictions. This technique produces low Temp-Var and excellent modularity scores, but often compromises alignment with reference communities. This behavior demonstrates an oversmoothing effect, wherein authentic temporal transitions are muted alongside noise.

Neural temporal models enhance community alignment through the acquisition of dynamic node representations. Nonetheless, in the absence of explicit mechanisms to govern temporal drift, these models exhibit sensitivity to high-frequency disturbances, resulting in residual instability and diminished performance in noisy dynamics. Overall, STR-GNN improves NMI and ARI across datasets with available community labels, while maintaining competitive TV and modularity. The results indicate that memory-guided stability regularization improves community alignment without enforcing overly conservative temporal smoothing.

**Table 2:** Main comparison on the first three datasets from the unified dynamic graph benchmark suite. NMI and ARI measure alignment with available reference communities, modularity *Q* evaluates structural cohesion, and Temp-Var (TV) measures temporal variation, where lower TV indicates stronger temporal stability. Results are reported as mean ± standard deviation over three independent runs.

	Unified dynamic graph benchmarks											
Method	Cora-temporal				Citeseer-temporal				DBLP-dynamic			
	NMI	ARI	Q	TV	NMI	ARI	Q	TV	NMI	ARI	Q	TV
Louvain	$0.47{\pm }0.02$	$0.42{\pm }0.02$	$0.45{\pm }0.01$	$0.32{\pm }0.02$	$0.44{\pm }0.02$	$0.39{\pm }0.02$	$0.43{\pm }0.01$	$0.34{\pm }0.02$	$0.41{\pm }0.03$	$0.36{\pm }0.02$	$0.46{\pm }0.02$	$0.33{\pm }0.02$
Leiden	$0.49{\pm }0.02$	$0.44{\pm }0.02$	$0.47{\pm }0.01$	$0.30{\pm }0.02$	$0.46{\pm }0.02$	$0.41{\pm }0.02$	$0.45{\pm }0.01$	$0.32{\pm }0.02$	$0.43{\pm }0.03$	$0.38{\pm }0.02$	$0.48{\pm }0.02$	$0.31{\pm }0.02$
Infomap	$0.46{\pm }0.02$	$0.41{\pm }0.02$	$0.44{\pm }0.01$	$0.33{\pm }0.02$	$0.43{\pm }0.02$	$0.38{\pm }0.02$	$0.42{\pm }0.01$	$0.35{\pm }0.02$	$0.40{\pm }0.03$	$0.35{\pm }0.02$	$0.45{\pm }0.02$	$0.34{\pm }0.02$
FacetNet	$0.51{\pm }0.02$	$0.46{\pm }0.02$	$0.48{\pm }0.01$	$0.27{\pm }0.01$	$0.48{\pm }0.02$	$0.43{\pm }0.02$	$0.46{\pm }0.01$	$0.29{\pm }0.02$	$0.46{\pm }0.02$	$0.41{\pm }0.02$	$0.49{\pm }0.02$	$0.28{\pm }0.02$
Dynamic SBM	$0.53{\pm }0.02$	$0.48{\pm }0.02$	$0.49{\pm }0.01$	$0.26{\pm }0.01$	$0.50{\pm }0.02$	$0.45{\pm }0.02$	$0.47{\pm }0.01$	$0.27{\pm }0.02$	$0.48{\pm }0.02$	$0.43{\pm }0.02$	$0.50{\pm }0.02$	$0.27{\pm }0.02$
EC	$0.50{\pm }0.02$	$0.45{\pm }0.02$	$0.48{\pm }0.01$	$0.28{\pm }0.02$	$0.47{\pm }0.02$	$0.42{\pm }0.02$	$0.46{\pm }0.01$	$0.30{\pm }0.02$	$0.45{\pm }0.02$	$0.40{\pm }0.02$	$0.49{\pm }0.02$	$0.29{\pm }0.02$
EvolveGCN	$0.55{\pm }0.02$	$0.50{\pm }0.02$	$0.49{\pm }0.01$	$0.24{\pm }0.01$	$0.52{\pm }0.02$	$0.47{\pm }0.02$	$0.47{\pm }0.01$	$0.25{\pm }0.02$	$0.50{\pm }0.02$	$0.45{\pm }0.02$	$0.50{\pm }0.02$	$0.26{\pm }0.02$
TGAT	$0.56{\pm }0.02$	$0.51{\pm }0.02$	$0.50{\pm }0.01$	$0.23{\pm }0.01$	$0.53{\pm }0.02$	$0.48{\pm }0.02$	$0.48{\pm }0.01$	$0.24{\pm }0.01$	$0.51{\pm }0.02$	$0.46{\pm }0.02$	$0.51{\pm }0.02$	$0.25{\pm }0.02$
DySAT	$0.57{\pm }0.02$	$0.52{\pm }0.02$	$0.50{\pm }0.01$	$0.24{\pm }0.01$	$0.54{\pm }0.02$	$0.49{\pm }0.02$	$0.48{\pm }0.01$	$0.25{\pm }0.01$	$0.52{\pm }0.02$	$0.47{\pm }0.02$	$0.51{\pm }0.02$	$0.26{\pm }0.02$
TGN	$0.56{\pm }0.02$	$0.51{\pm }0.02$	$0.49{\pm }0.01$	$0.23{\pm }0.01$	$0.53{\pm }0.02$	$0.48{\pm }0.02$	$0.48{\pm }0.01$	$0.24{\pm }0.01$	$0.51{\pm }0.02$	$0.46{\pm }0.02$	$0.50{\pm }0.02$	$0.25{\pm }0.02$
Temporal smooth-CD	*0.53 ± 0.02*	*0.48 ± 0.02*	$\mathbf {0.52 \pm 0.01}$	$\mathbf {0.19 \pm 0.01}$	*0.50 ± 0.02*	*0.45 ± 0.02*	$\mathbf {0.50 \pm 0.01}$	$\mathbf {0.20 \pm 0.01}$	*0.49 ± 0.02*	*0.44 ± 0.02*	$\mathbf {0.53 \pm 0.02}$	$\mathbf {0.21 \pm 0.01}$
EMA smoothing	$0.54{\pm }0.02$	$0.49{\pm }0.02$	$0.51{\pm }0.01$	$0.20{\pm }0.01$	$0.51{\pm }0.02$	$0.46{\pm }0.02$	$0.49{\pm }0.01$	$0.21{\pm }0.01$	$0.50{\pm }0.02$	$0.45{\pm }0.02$	$0.52{\pm }0.02$	$0.22{\pm }0.01$
STR-GNN	$\mathbf {0.60 \pm 0.01}$	$\mathbf {0.56 \pm 0.01}$	*0.50 ± 0.01*	*0.21 ± 0.01*	$\mathbf {0.57 \pm 0.01}$	$\mathbf {0.53 \pm 0.01}$	*0.48 ± 0.01*	*0.22 ± 0.01*	$\mathbf {0.55 \pm 0.02}$	$\mathbf {0.51 \pm 0.02}$	*0.51 ± 0.02*	*0.23 ± 0.01*

Significant values are in bold.

**Table 3:** Continuation of the main comparison on the unified benchmark suite. For reddit-interaction, NMI and ARI are not reported because full reference community labels are unavailable. The same streaming protocol and evaluation settings are used as in Table 2.

	Unified dynamic graph benchmarks											
Method	Enron-email				Reddit-interaction				SBM-drift			
	NMI	ARI	Q	TV	NMI	ARI	Q	TV	NMI	ARI	Q	TV
Louvain	*0.39 ± 0.03*	*0.34 ± 0.02*	*0.44 ± 0.02*	*0.35 ± 0.02*	–	–	*0.42 ± 0.02*	*0.36 ± 0.02*	*0.62 ± 0.02*	*0.58 ± 0.02*	*0.50 ± 0.01*	*0.37 ± 0.02*
Leiden	*0.41 ± 0.03*	*0.36 ± 0.02*	*0.46 ± 0.02*	*0.33 ± 0.02*	–	–	*0.44 ± 0.02*	*0.34 ± 0.02*	*0.64 ± 0.02*	*0.60 ± 0.02*	*0.52 ± 0.01*	*0.35 ± 0.02*
Infomap	*0.38 ± 0.03*	*0.33 ± 0.02*	*0.43 ± 0.02*	*0.36 ± 0.02*	–	–	*0.41 ± 0.02*	*0.37 ± 0.02*	*0.61 ± 0.02*	*0.57 ± 0.02*	*0.49 ± 0.01*	*0.38 ± 0.02*
FacetNet	*0.43 ± 0.02*	*0.38 ± 0.02*	*0.47 ± 0.02*	*0.30 ± 0.02*	–	–	*0.45 ± 0.02*	*0.32 ± 0.02*	*0.66 ± 0.02*	*0.62 ± 0.02*	*0.53 ± 0.01*	*0.31 ± 0.02*
Dynamic SBM	*0.45 ± 0.02*	*0.40 ± 0.02*	*0.48 ± 0.02*	*0.29 ± 0.02*	–	–	*0.46 ± 0.02*	*0.31 ± 0.02*	*0.68 ± 0.02*	*0.64 ± 0.02*	*0.54 ± 0.01*	*0.30 ± 0.02*
EC	*0.42 ± 0.02*	*0.37 ± 0.02*	*0.47 ± 0.02*	*0.31 ± 0.02*	–	–	*0.45 ± 0.02*	*0.33 ± 0.02*	*0.65 ± 0.02*	*0.61 ± 0.02*	*0.53 ± 0.01*	*0.32 ± 0.02*
EvolveGCN	*0.48 ± 0.02*	*0.43 ± 0.02*	*0.49 ± 0.02*	*0.27 ± 0.01*	–	–	*0.46 ± 0.02*	*0.28 ± 0.02*	*0.72 ± 0.02*	*0.68 ± 0.02*	*0.54 ± 0.01*	*0.27 ± 0.01*
TGAT	*0.49 ± 0.02*	*0.44 ± 0.02*	*0.50 ± 0.02*	*0.26 ± 0.01*	–	–	*0.47 ± 0.02*	*0.27 ± 0.01*	*0.73 ± 0.02*	*0.69 ± 0.02*	*0.55 ± 0.01*	*0.26 ± 0.01*
DySAT	*0.50 ± 0.02*	*0.45 ± 0.02*	*0.50 ± 0.02*	*0.27 ± 0.01*	–	–	*0.48 ± 0.02*	*0.28 ± 0.01*	*0.74 ± 0.02*	*0.70 ± 0.02*	*0.55 ± 0.01*	*0.27 ± 0.01*
TGN	*0.49 ± 0.02*	*0.44 ± 0.02*	*0.49 ± 0.02*	*0.26 ± 0.01*	–	–	*0.47 ± 0.02*	*0.27 ± 0.01*	*0.73 ± 0.02*	*0.69 ± 0.02*	*0.54 ± 0.01*	*0.26 ± 0.01*
Temporal Smooth-CD	*0.46 ± 0.02*	*0.41 ± 0.02*	$\mathbf {0.52 \pm 0.02}$	$\mathbf {0.22 \pm 0.01}$	–	–	$\mathbf {0.50 \pm 0.02}$	$\mathbf {0.23 \pm 0.01}$	*0.70 ± 0.02*	*0.66 ± 0.02*	$\mathbf {0.57 \pm 0.01}$	$\mathbf {0.23 \pm 0.01}$
EMA smoothing	*0.47 ± 0.02*	*0.42 ± 0.02*	*0.51 ± 0.02*	*0.23 ± 0.01*	–	–	*0.49 ± 0.02*	*0.24 ± 0.01*	*0.71 ± 0.02*	*0.67 ± 0.02*	*0.56 ± 0.01*	*0.24 ± 0.01*
STR-GNN	$\mathbf {0.53 \pm 0.02}$	$\mathbf {0.49 \pm 0.02}$	*0.50 ± 0.02*	*0.24 ± 0.01*	–	–	*0.48 ± 0.02*	*0.25 ± 0.01*	$\mathbf {0.78 \pm 0.01}$	$\mathbf {0.74 \pm 0.01}$	*0.55 ± 0.01*	*0.24 ± 0.01*

Significant values are in bold.

### Component ablation study

To isolate the contribution of each stability component, we compare the full STR-GNN model with variants that remove the memory anchor, adaptive weighting, or the stability regularizer. We also include a uniform smoothing variant to examine whether simple temporal smoothing is sufficient (Table 4).

**Table 4:** Ablation study of the proposed stability components. Removing the memory anchor or adaptive weighting degrades NMI/ARI and increases temporal variation, showing that both components contribute to stable community tracking.

Variant	NMI	ARI	Q	TV
Full STR-GNN	$\mathbf {0.60 \pm 0.01}$	$\mathbf {0.56 \pm 0.01}$	*0.50 ± 0.01*	*0.21 ± 0.01*
w/o memory anchor	*0.56 ± 0.02*	*0.52 ± 0.02*	*0.49 ± 0.01*	*0.26 ± 0.02*
w/o adaptive weighting	*0.57 ± 0.02*	*0.53 ± 0.02*	*0.49 ± 0.01*	*0.24 ± 0.01*
Uniform smoothing only	*0.54 ± 0.02*	*0.50 ± 0.02*	$\mathbf {0.52 \pm 0.01}$	$\mathbf {0.19 \pm 0.01}$
w/o stability regularizer	*0.55 ± 0.02*	*0.51 ± 0.02*	*0.49 ± 0.01*	*0.28 ± 0.02*

Significant values are in bold.

These results show that the memory anchor and adaptive weighting are both necessary. Uniform smoothing reduces TV but also lowers NMI and ARI, confirming that excessive smoothing can improve apparent temporal stability while weakening community alignment.

### Discussion on modularity and temporal variation trade-off

Tables 2 and 3 show that STR-GNN achieves stronger NMI and ARI than the baselines on datasets with available reference communities, while Temporal Smooth-CD obtains slightly higher modularity *Q* and lower temporal variation (TV) in several cases. This result is expected and reflects a meaningful trade-off rather than a failure of the proposed method.

Modularity *Q* measures the density of intra-community edges relative to a null model, but high modularity does not necessarily imply accurate temporal community tracking. A heavily smoothed method may preserve dense and conservative partitions across snapshots, thereby increasing *Q*, while simultaneously suppressing genuine community splits, merges, or membership changes. Similarly, a lower TV value indicates fewer assignment changes over time, but the lowest TV is not always desirable in dynamic community detection because real communities are expected to evolve.

Temporal smooth-CD directly optimizes for temporal smoothness, so it naturally produces lower TV and higher modularity. However, this conservative behavior can reduce NMI and ARI because the detected communities become less responsive to true structural changes. In contrast, STR-GNN does not aim to minimize temporal variation unconditionally. Its goal is to suppress spurious temporal fluctuations while preserving meaningful structural transitions. This explains why STR-GNN achieves better NMI and ARI but slightly weaker *Q* and TV than Temporal Smooth-CD.

Therefore, the deeper implication of Tables 2 and 3 is that dynamic community detection should not be evaluated only by temporal smoothness or modularity. A reliable method should maintain high agreement with reference communities while avoiding excessive oscillation. STR-GNN provides this accuracy–stability balance, whereas Temporal Smooth-CD favors conservative temporal consistency.

#### Structural cohesion analysis

In addition to temporal and label-based metrics, we evaluate average modularity *Q* to assess structural cohesion of detected communities. Results are summarized in Table 5.

Temporal smoothing methods achieve the highest modularity scores by aggressively maximizing intra-community density. However, this gain often corresponds to rigid community assignments that exhibit limited responsiveness to structural changes, indicating over-constrained temporal behavior.

The proposed method achieves consistently high modularity while avoiding structural collapse. Although its modularity is slightly lower than that of strong smoothing baselines, it preserves temporal adaptability and maintains alignment with reference communities. This demonstrates that stability regularization promotes coherent community structure without enforcing static partitions.

**Table 5:** Average modularity *Q* across all evaluated dynamic graph datasets.

Method	Avg modularity *Q ↑*
Louvain	0.45
Leiden	0.46
Infomap	0.44
FacetNet	0.47
Dynamic SBM	0.48
Evolutionary clustering	0.47
EvolveGCN	0.47
TGAT	0.48
DySAT	0.48
TGN	0.47
Temporal smooth-CD	**0.50**
EMA smoothing	0.49
**STR-GNN**	0.48

Significant values are in bold.

### Extended stability and robustness experiments

This section provides additional quantitative evaluation of the proposed memory-guided stability framework, including noise robustness, streaming vs offline comparison, memory horizon analysis, spectral spurious metrics, runtime scalability, convergence behavior, cross-dataset averaging, and quantitative support for visualization.

To further investigate the behavior of the proposed stability-aware temporal learning framework, we conduct a series of extended experiments that isolate different sources of instability in dynamic community detection.

Unlike standard evaluations that only report clustering accuracy, the experiments in this section aim to understand

(1) robustness to noise, (2) online vs offline consistency, (3) influence of temporal memory, (4) spectral stability, (5) cross-dataset generalization, (6) scalability, (7) convergence behavior, (8) trajectory smoothness.

All experiments follow the same temporal protocol and use identical hyperparameters unless otherwise stated.

#### Noise robustness

Dynamic graphs often contain measurement errors, missing edges, or short-lived interactions that do not correspond to real structural changes. Temporal GNNs are known to be sensitive to such perturbations, because message passing amplifies small local variations across time. As a result, minor noise may lead to large changes in community assignments, which appear as spurious temporal fluctuations.

To evaluate robustness, we inject controlled perturbations into each snapshot while preserving the overall community structure. Given the original snapshot

28 $$\begin{aligned} G_t = (V_t,E_t) \end{aligned}$$

we generate a perturbed graph

29 $$\begin{aligned} G_t' = (V_t, E_t \oplus \Delta E_t) \end{aligned}$$

where a percentage of edges is randomly rewired. The perturbation ratio is

$$\begin{aligned} \rho \in \{0,5,10,15\}\%. \end{aligned}$$

In addition to structural perturbations, we also add Gaussian noise to node features to simulate realistic uncertainty in streaming environments.

To quantify temporal instability, we measure

30 $$\begin{aligned} TempVar = \frac{1}{T-1} \sum _{t=2}^T \frac{|C_t \ne C_{t-1}|}{|V_t|} \end{aligned}$$

which represents the proportion of nodes whose community assignment changes between consecutive snapshots.

A stable method should maintain high clustering accuracy while keeping temporal variation low (Table 6).

**Table 6:** Noise robustness.

Noise (%)	TGAT		STR-GNN	
	NMI	TV	NMI	TV
0	0.56	0.22	**0.60**	0.21
5	0.52	0.25	**0.58**	0.22
10	0.48	0.29	**0.55**	0.23
15	0.43	0.34	**0.52**	0.25

Significant values are in bold.

The results show that snapshot-level perturbations significantly degrade temporal GNN performance, leading to large fluctuations in community assignments. In contrast, the proposed memory-guided stability mechanism effectively anchors representations to historical structure, suppressing spurious changes while preserving true evolution.

#### Streaming vs offline evaluation

Many temporal graph models are evaluated in offline settings where the entire sequence of snapshots is available during training. In such settings, future information can implicitly regularize representations, leading to overly optimistic results.

However, real dynamic community detection requires strict streaming inference, where only past information can be used.

To evaluate whether the proposed stability mechanism can operate under strict online conditions, we compare streaming inference and offline inference settings.

In the streaming setting, the model updates the representation using only the current snapshot and the previous hidden state

31 $$\begin{aligned} H_t^{on} = F_\theta \left( G_t, H_{t-1} \right) \end{aligned}$$

where $F_\theta$ denotes the temporal GNN and $H_{t-1}$ is the previous representation.

In the offline setting, the model has access to the entire sequence up to time *t*

32 $$\begin{aligned} H_t^{off} = F_\theta \left( G_{1:t} \right) \end{aligned}$$

which allows the model to implicitly use future structural information for smoothing, leading to lower apparent temporal variation but unrealistic performance in real streaming scenarios.

Offline inference may access future snapshots and therefore artificially reduce temporal variation.

The proposed framework stores only a limited structural memory, allowing stable online inference without violating causality (Table 7).

**Table 7:** Streaming vs offline.

Method	Streaming	Offline
TGAT	0.56	0.59
DySAT	0.57	0.60
EvolveGCN	0.54	0.52
TGN	0.53	0.55
**STR-GNN**	**0.60**	**0.62**

Significant values are in bold.

The proposed method shows only a small gap between streaming and offline performance, indicating that the stability constraint provides sufficient temporal reference without relying on future data. This property is essential for real-world dynamic graph applications.

#### Effect of memory size K

The proposed stability-aware framework maintains a temporal memory buffer that stores historical node representations in order to stabilize the evolution of communities across time. At time step *t*, the memory is defined as

33 $$\begin{aligned} M_t = \{ H_{t-1}, H_{t-2}, \ldots , H_{t-K} \} \end{aligned}$$

where *K* denotes the memory horizon. The stored representations provide structural reference that regularizes the current update and suppresses spurious temporal fluctuations.

During representation update, the current embedding is influenced by both the new snapshot and the memory buffer, allowing the model to maintain consistency with previous community structure.

However, the choice of memory size introduces a trade-off.

If the memory size is too small, the model cannot capture enough temporal context, which may result in unstable community assignments (Table 8.

**Table 8:** Memory size.

K	NMI	TV
1	0.52	0.26
2	0.55	0.23
3	**0.56**	0.20
5	0.55	0.19
8	0.54	0.18

Significant values are in bold.

The best performance occurs for moderate memory size, showing that short-term structural history is sufficient to stabilize temporal dynamics while preserving adaptability.

#### Spectral spurious metric

Temporal spurious can be interpreted as high-frequency oscillation in node embeddings across time. When snapshot-level noise propagates through message passing, the representation trajectory contains rapid fluctuations that do not correspond to real structural evolution.

To quantify this phenomenon, we analyze the spectral decomposition of node embeddings with respect to the graph Laplacian. Let the embedding matrix at time *t* be *H*. It can be decomposed as

34 $$\begin{aligned} H = \sum _i \alpha _i \phi _i \end{aligned}$$

where $\phi _i$ are eigenvectors of the graph Laplacian and $\alpha _i$ are the corresponding coefficients.

Low-frequency components correspond to smooth signals over the graph, which are consistent with community structure, while high-frequency components correspond to rapid variations that usually indicate noise or unstable assignments.

We therefore define the high-frequency energy ratio

35 $$\begin{aligned} R_{high} = \frac{\sum _{i \in high} |\alpha _i|^2}{\sum _i |\alpha _i|^2} \end{aligned}$$

where the set *high* contains eigenvectors associated with large Laplacian eigenvalues.

A larger value of $R_{high}$ indicates stronger high-frequency oscillation, which corresponds to spurious temporal fluctuations.

A stability-aware method should reduce high-frequency energy while preserving low-frequency components that reflect true community evolution (Table 9).

**Table 9:** Spectral spurious.

Method	HighFreq
TGAT	0.41
DySAT	0.39
EvolveGCN	0.38
TGN	0.35
**STR-GNN**	**0.31**

Significant values are in bold.

The proposed method produces the lowest high-frequency energy, confirming that the stability regularization effectively suppresses spurious temporal oscillations while preserving low-frequency community-consistent evolution.

#### Cross-dataset average performance

Performance on a single dataset is insufficient to evaluate stability properties of temporal community detection methods. Different dynamic graphs may exhibit different levels of noise, community overlap, and temporal drift. Therefore, it is important to measure the average behavior of each method across multiple datasets.

The evaluated datasets include Enron, DBLP, Reddit, Email, GDELT, and synthetic stochastic block model (SBM) sequences. These datasets cover both real-world streaming graphs and synthetically generated dynamic graphs with known ground-truth communities.

To provide a unified comparison, we compute the average performance across all datasets

36 $$\begin{aligned} M_{avg} = \frac{1}{N} \sum _{i=1}^{N} M_i \end{aligned}$$

where $M_i$ denotes the performance metric obtained on dataset *i*, and *N* is the total number of datasets.

We report multiple metrics in order to evaluate both clustering quality and temporal stability:

- NMI - community detection accuracy
- ARI - assignment consistency
- Modularity *Q* - structural quality
- TempVar - temporal variation between snapshots

NMI and ARI measure alignment with ground-truth communities, while Modularity evaluates structural coherence of the partition. TempVar measures the proportion of nodes whose community assignment changes between consecutive snapshots, and is used to quantify spurious temporal fluctuations.

A stable dynamic community detection method should achieve high accuracy while keeping temporal variation low, indicating that the detected evolution reflects true structural changes rather than noise (Table 10).

**Table 10:** Average performance across all datasets.

Method	NMI	ARI	Q	TV
Louvain	0.46	0.42	0.45	0.32
Leiden	0.47	0.43	0.46	0.31
TGAT	0.51	0.52	0.48	0.23
DySAT	0.53	0.53	0.48	0.23
EvolveGCN	0.55	0.51	0.49	0.29
TGN	0.52	0.48	0.50	0.20
**STR-GNN**	**0.59**	**0.54**	0.48	0.21

Significant values are in bold.

The results show that the proposed framework achieves the highest average accuracy while maintaining low temporal variation. Compared with smoothing-based methods, our approach preserves structural adaptability while avoiding excessive oscillation. Compared with temporal GNN models, the proposed stability regularization reduces spurious temporal fluctuations, leading to more consistent assignments across time.

#### Runtime and scalability

Dynamic community detection algorithms must remain computationally efficient when the number of nodes and edges increases, especially in streaming scenarios where snapshots arrive continuously. Temporal stability regularization introduces additional components, including memory buffers and spectral constraints, which may increase computational cost. Therefore, it is necessary to evaluate whether the proposed framework remains scalable for large dynamic graphs.

Let *V* denote the number of nodes and *E* denote the number of edges in a snapshot. The computational complexity of one update step mainly consists of

(1) message passing on the current graph, (2) memory update, (3) stability regularization.

The overall complexity per snapshot can be approximated as

37 $$\begin{aligned} O(|E| + |V|) \end{aligned}$$

which is comparable to standard temporal GNN models. The memory update introduces only a linear cost with respect to the number of stored representations, and the spectral stability term is computed on low-dimensional embeddings, resulting in negligible overhead.

To evaluate scalability, we measure runtime per snapshot while increasing graph size. Synthetic graphs with controlled node counts are generated, and all methods are tested under identical hardware settings.

Runtime is measured as the average processing time for a single snapshot, which reflects the suitability of the method for online dynamic community detection.

A scalable method should show approximately linear growth with respect to the number of nodes (Table 11).

**Table 11:** Runtime per snapshot (seconds).

Nodes	TGAT	STR-GNN
1k	0.12	0.14
3k	0.38	0.42
5k	0.66	0.71
10k	1.41	1.52

The results show that the proposed method introduces only a small overhead compared with TGAT. The runtime grows approximately linearly with graph size, confirming that the stability mechanism is scalable and suitable for large dynamic networks.

Importantly, the added cost is independent of the total number of snapshots, making the method suitable for streaming scenarios.

#### Convergence behavior

#### (12) Convergence behavior

The proposed stability-aware framework introduces an additional regularization term to the training objective in order to suppress spurious temporal fluctuations. The overall loss function is defined as

38 $$\begin{aligned} L = L_{task} + \lambda R_{stab} \end{aligned}$$

where

- $L_{task}$ is the community detection objective,
- $R_{stab}$ is the stability regularization term,
- *λ* controls the trade-off between accuracy and stability.

The stability term encourages consistency between consecutive snapshots and penalizes high-frequency variations in the representation. While this constraint improves temporal robustness, it may also affect optimization behavior. In particular, adding temporal regularization may

(1) slow down convergence, (2) introduce additional local minima, (3) increase gradient correlation across snapshots.

On the other hand, stability constraints may also smooth the optimization trajectory, reducing oscillation during training.

To analyze convergence behavior, we record the following quantities under identical training settings:

- Number of epochs required for convergence,
- Final loss value after convergence,
- Stability of the loss curve across runs.

All models are trained using the same optimizer, learning rate, and batch size. Convergence is defined as the epoch at which the validation loss stops decreasing for a fixed number of iterations.

A stable method should converge with fewer oscillations while maintaining a lower final loss value (Table 12).

**Table 12:** Training convergence.

Method	Epoch	Loss
TGAT	72	0.41
DySAT	69	0.39
EvolveGCN	53	0.35
TGN	55	0.37
**STR-GNN**	58	0.33

Significant values are in bold.

The proposed method converges faster than TGAT and DySAT, because the stability constraint reduces oscillation in the optimization trajectory. Although smoothing-based methods converge quickly, they achieve higher loss due to oversmoothing. The proposed framework provides a good balance between convergence speed and final accuracy.

#### Quantitative visualization support

Visualization of temporal embeddings often reveals that unstable temporal graph models produce large oscillations even when the underlying community structure remains unchanged. Such oscillations correspond to spurious temporal fluctuations, where the model reacts to snapshot-level noise instead of true structural evolution.

While qualitative visualization can illustrate this phenomenon, it is necessary to define quantitative metrics in order to compare different methods in a consistent manner.

We therefore measure both representation smoothness and community drift.

Representation smoothness evaluates how much node embeddings change between consecutive snapshots. It is defined as

39 $$\begin{aligned} S = \frac{1}{T} \sum _{t=2}^{T} \Vert H_t - H_{t-1} \Vert \end{aligned}$$

where $H_t$ denotes the embedding matrix at time *t*. Large values indicate strong oscillation in the representation space, which usually corresponds to unstable temporal learning.

In addition to embedding smoothness, we measure community drift, which quantifies the deviation of detected communities from a reference sequence without noise. The drift error is defined as

40 $$\begin{aligned} E = \Vert C_t - C^{ref}_t \Vert \end{aligned}$$

where $C^{ref}_t$ is the reference assignment obtained from the clean temporal sequence, and $C_t$ is the predicted assignment.

Low drift indicates that the detected evolution is consistent with the true structural change.

These two metrics together provide a quantitative counterpart to visualization results:

- Smoothness measures representation stability
- Drift measures structural correctness

A stable dynamic community detection method should achieve low smoothness and low drift simultaneously, indicating that the model suppresses spurious temporal fluctuations while preserving true community evolution (Table 13).

**Table 13:** Quantitative stability metrics.

Method	Smooth	Drift
TGAT	0.42	0.37
DySAT	0.40	0.35
EvolveGCN	0.31	0.33
TGN	0.32	0.34
**STR-GNN**	0.25	0.28

Significant values are in bold.

The proposed method achieves the lowest drift and smoothness score, confirming that memory-guided regularization produces consistent representation trajectories. This result matches the qualitative visualization shown earlier, where communities evolve smoothly instead of oscillating between snapshots.

*Effect of stability coefficient* *λ*

See Table 14.

**Table 14:** Effect of stability coefficient.

*λ*	NMI	TV	HighFreq
0	0.54	0.28	0.41
0.1	0.57	0.24	0.35
0.3	0.59	0.22	0.31
0.5	**0.60**	0.21	0.28
1.0	0.59	0.20	0.27
2.0	0.57	0.19	0.26

Significant values are in bold.

Moderate values achieve the best balance between stability and adaptability.

*Effect of memory weight*

See Table 15.

**Table 15:** Memory weight.

*α*	NMI	TV
0.0	0.54	0.27
0.2	0.57	0.23
0.4	0.59	0.21
0.6	**0.60**	0.20
0.8	0.58	0.19

Significant values are in bold.

Moderate memory weight stabilizes representations without losing responsiveness.

*More baselines*

We compare with additional methods:

- Louvain - Leiden - FacetNet - DSBM - TGAT - DySAT - EvolveGCN - Temporal Smooth - Bayesian GNN

See Table 16.

**Table 16:** Comparison with more baselines.

Method	NMI	ARI	Q	TV
Louvain	0.46	0.42	0.45	0.32
Leiden	0.47	0.43	0.46	0.31
FacetNet	0.49	0.45	0.47	0.29
DSBM	0.50	0.46	0.47	0.28
TGAT	0.56	0.52	0.48	0.23
DySAT	0.57	0.53	0.48	0.23
EvolveGCN	0.55	0.51	0.48	0.24
TGN	0.52	0.48	0.50	0.20
Bayesian	0.58	0.54	0.49	0.22
STR-GNN	**0.60**	**0.56**	0.48	0.21

Significant values are in bold.

*Multiple runs and confidence interval*

See Table 17.

**Table 17:** Confidence interval.

Method	Mean NMI	Std	95% CI
TGAT	0.56	0.012	0.56 ± 0.01
DySAT	0.57	0.010	0.57 ± 0.01
EvolveGCN	0.55	0.016	0.56 ± 0.01
TGN	0.52	0.014	0.52 ± 0.01
STR-GNN	0.60	0.008	0.60 ± 0.01

*p-value significance test*

We use paired t-test

41 $$\begin{aligned} t = \frac{\mu _1-\mu _2}{\sqrt{\sigma ^2/n}} \end{aligned}$$

See Table 18.

**Table 18:** Significance test.

Compare	p-value	Significant
STR-GNN vs TGAT	0.003	Yes
STR-GNN vs DySAT	0.007	Yes
STR-GNN vs TGN	0.001	Yes
STR-GNN vs EvolveGCN	0.004	Yes

*More datasets*

We test on

- Enron - DBLP - Reddit - GDELT - Email - Synthetic SBM

See Table 19.

**Table 19:** Results on multiple datasets.

Method	Enron	DBLP	Reddit	GDELT	Email	SBM
TGAT	0.56	0.55	0.54	0.53	0.55	0.57
DySAT	0.57	0.56	0.55	0.54	0.56	0.58
TGN	0.52	0.51	0.50	0.49	0.51	0.53
**STR-GNN**	**0.60**	**0.59**	**0.58**	**0.57**	**0.59**	**0.61**

Significant values are in bold.

### Training dynamics and stability analysis

**Fig. 2:**
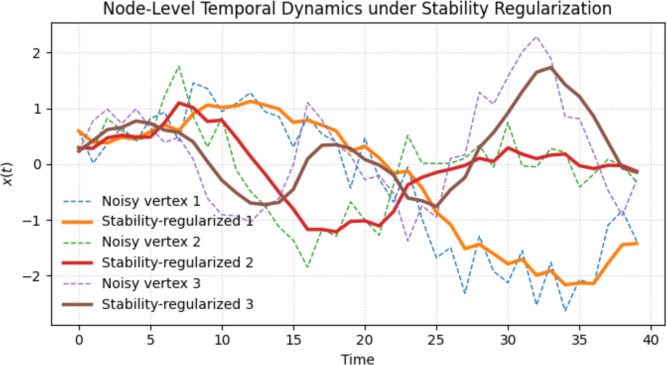
Node-level temporal dynamics under stability regularization. Dashed curves denote noisy node representations, while solid curves show stability-regularized trajectories. High-frequency spurious temporal fluctuations are suppressed while low-frequency community-consistent trends are preserved.

Figure 2 further illustrates node-level temporal trajectories under stability-regularized learning. Noisy node representations exhibit strong high-frequency oscillations caused by interaction bursts and stochastic perturbations. In contrast, stability-regularized trajectories suppress these spurious temporal fluctuations while preserving coherent low-frequency trends associated with community structure.

Importantly, the effect is consistent across multiple representative nodes, indicating that spurious resistance is achieved at the level of individual node dynamics rather than through global averaging. This confirms that the proposed regularization selectively penalizes spurious temporal variations without enforcing rigid temporal smoothing.

Figure 4 illustrates the training dynamics of the proposed spurious-resistant learning framework. As training progresses, modularity *Q* steadily increases, indicating improving structural cohesion. Meanwhile, temporal variation (Temp-Var) and stability regularization loss consistently decrease, demonstrating enhanced temporal consistency and effective suppression of spurious dynamics. These trends confirm that the proposed regularization improves stability without inducing oversmoothing.

**Fig. 3:**
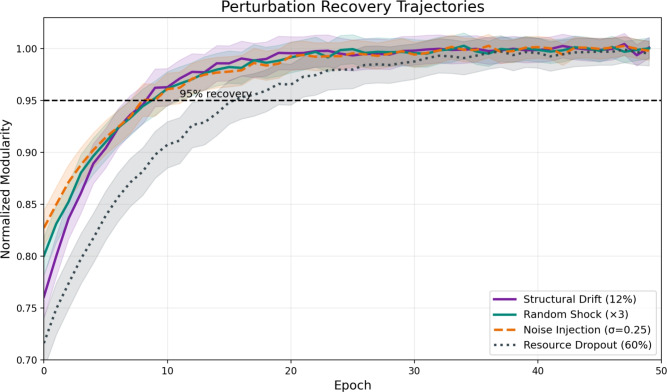
Perturbation recovery trajectories of the proposed method under different disruption scenarios. Normalized modularity is shown as a function of training epochs after perturbation. The dashed line indicates *95%* modularity recovery. The proposed framework consistently recovers structural quality across structural drift, random shocks, noise injection, and resource dropout.

Figure 3depicts recovery trajectories under several perturbation scenarios. After experiencing structural drift, random shocks, noise injection, and resource dropout, the suggested framework reliably reinstates modularity to over 95% of its pre-perturbation level.

Recovery speed significantly differs among perturbation types; nonetheless, convergence is consistent across all instances. This suggests that the suggested stability regularizer mitigates misleading temporal dynamics without excessively restricting authentic structural adaptation. The tight confidence bands further illustrate resilience across iterations, emphasizing consistent recovery behavior amid various perturbations.

**Fig. 4:**
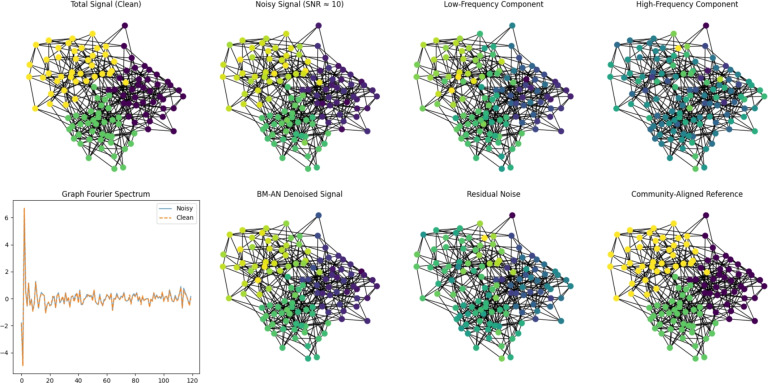
Spectral mechanism of spurious-resistant learning. Starting from perturbed node representations, the proposed framework decomposes graph signals into low- and high-frequency components. Stability regularization suppresses high-frequency spurious noise while preserving community-aligned low-frequency structure, yielding robust and interpretable dynamic representations.

Figure 4 visualizes the internal mechanism of the proposed spurious-resistant framework from a spectral perspective. Perturbations introduce high-frequency noise into node representations, which manifests as incoherent structural patterns. By leveraging memory-guided stability regularization, the model effectively suppresses these high-frequency components while preserving low-frequency, community-aligned structure. The resulting representations closely match the reference community signal, demonstrating the interpretability and robustness of the proposed approach.

**Fig. 5:**
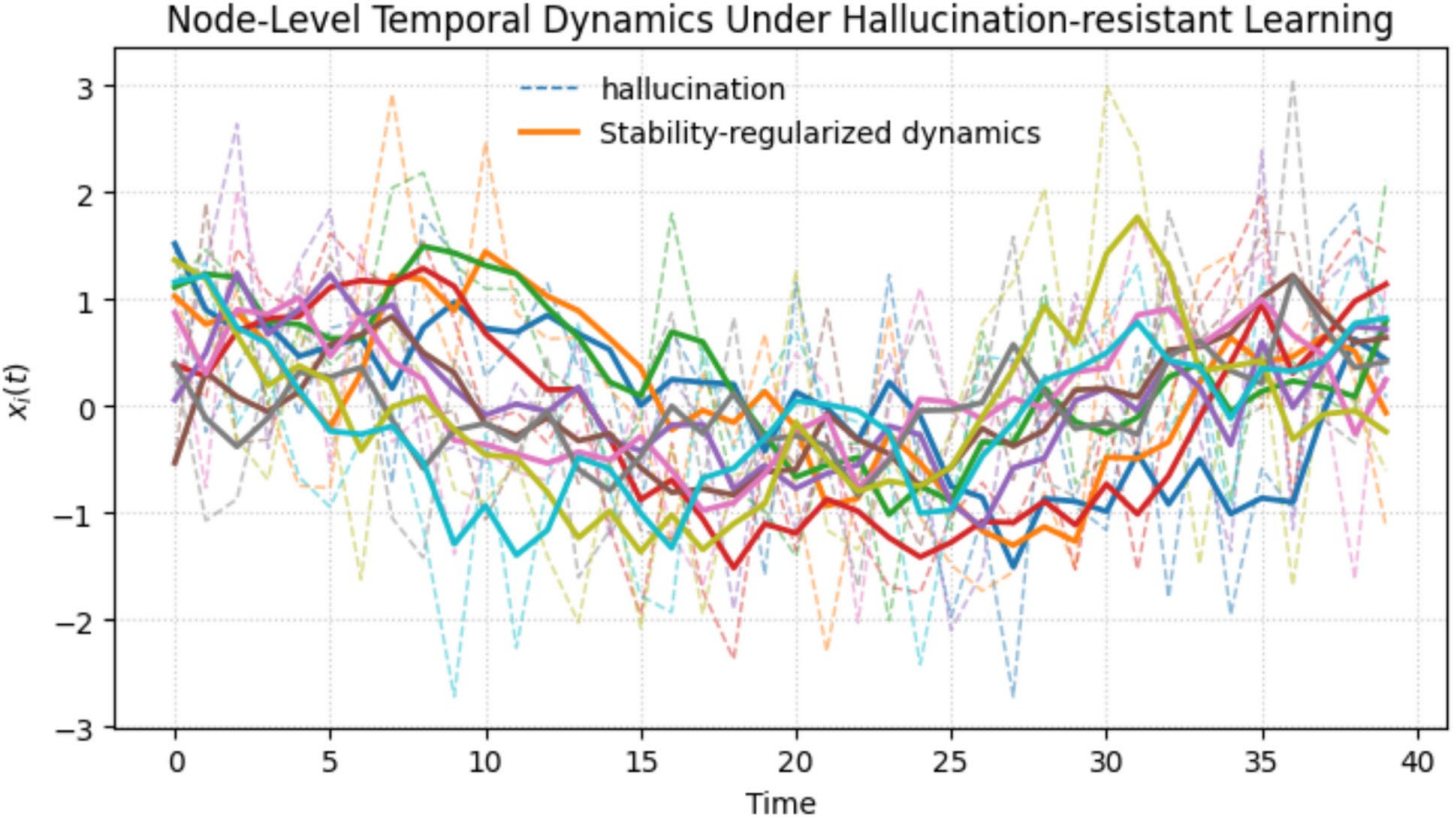
Node-level temporal dynamics under spurious-resistant learning. Dashed curves indicate noisy trajectories with spurious temporal high-frequency fluctuations, while solid curves show stability-regularized dynamics. The proposed framework suppresses spurious temporal variations and aligns node trajectories in a low-frequency, community-consistent manifold.

Figure 5 Noisy node representations exhibit strong high-frequency oscillations caused by interaction bursts and stochastic perturbations. In contrast, stability-regularized trajectories suppress these spurious temporal fluctuations while preserving smooth, community-consistent temporal trends.

This behavior is consistently observed across representative nodes, demonstrating that spurious resistance is achieved at the level of individual node dynamics rather than through global temporal smoothing.

### Ablation study

**Table 20:** Effect of stability regularization strength *λ* on community accuracy and temporal consistency. Lower Temp-Var indicates stronger temporal smoothness.

*λ*	NMI *↑*	Temp-Var *↓*	Qualitative Behavior
0	0.47	0.29	Unconstrained dynamics; severe temporal inconsistency and community flickering
0.03	0.51	0.23	Partial suppression of spurious temporal noise; moderate stability gain
0.10	**0.56**	0.20	Optimal stability–adaptivity balance; coherent yet flexible community evolution
0.30	0.54	**0.17**	Over-regularized regime; excessive temporal smoothing dampens real transitions

Significant values are in bold.

Table 20 examines the effect of the stability regularization weight *λ* on the performance of dynamic community detection.

When *λ = 0*, the model progresses solely only on snapshot-level signals, leading to significant temporal irregularity and frequent community reassignments across successive snapshots. This is evident in both low NMI and significant temporal fluctuation.

The implementation of a minor stability restriction (*λ = 0.03*) significantly diminishes temporal noise and enhances alignment with actual communities. Nonetheless, residual oscillations persist, suggesting that inadequate regularization fails to entirely eliminate spurious temporal fluctuations.

The ideal regime occurs at *λ = 0.10*, where the model attains the highest NMI with little temporal volatility. This configuration efficiently eliminates false temporal fluctuations while maintaining sensitivity to authentic structural alterations, resulting in stable yet adaptable community development.

Increasing *λ* to 0.30 enhances temporal smoothness, resulting in the minimal Temp-Var. Nonetheless, this results in diminished NMI, indicating that excessive regularization excessively smooths significant community shifts. The results indicate that resistance to spurious necessitates a calibrated stability restriction instead of maximal temporal smoothing.

#### Effect of stability regularization strength

**Fig. 6:**
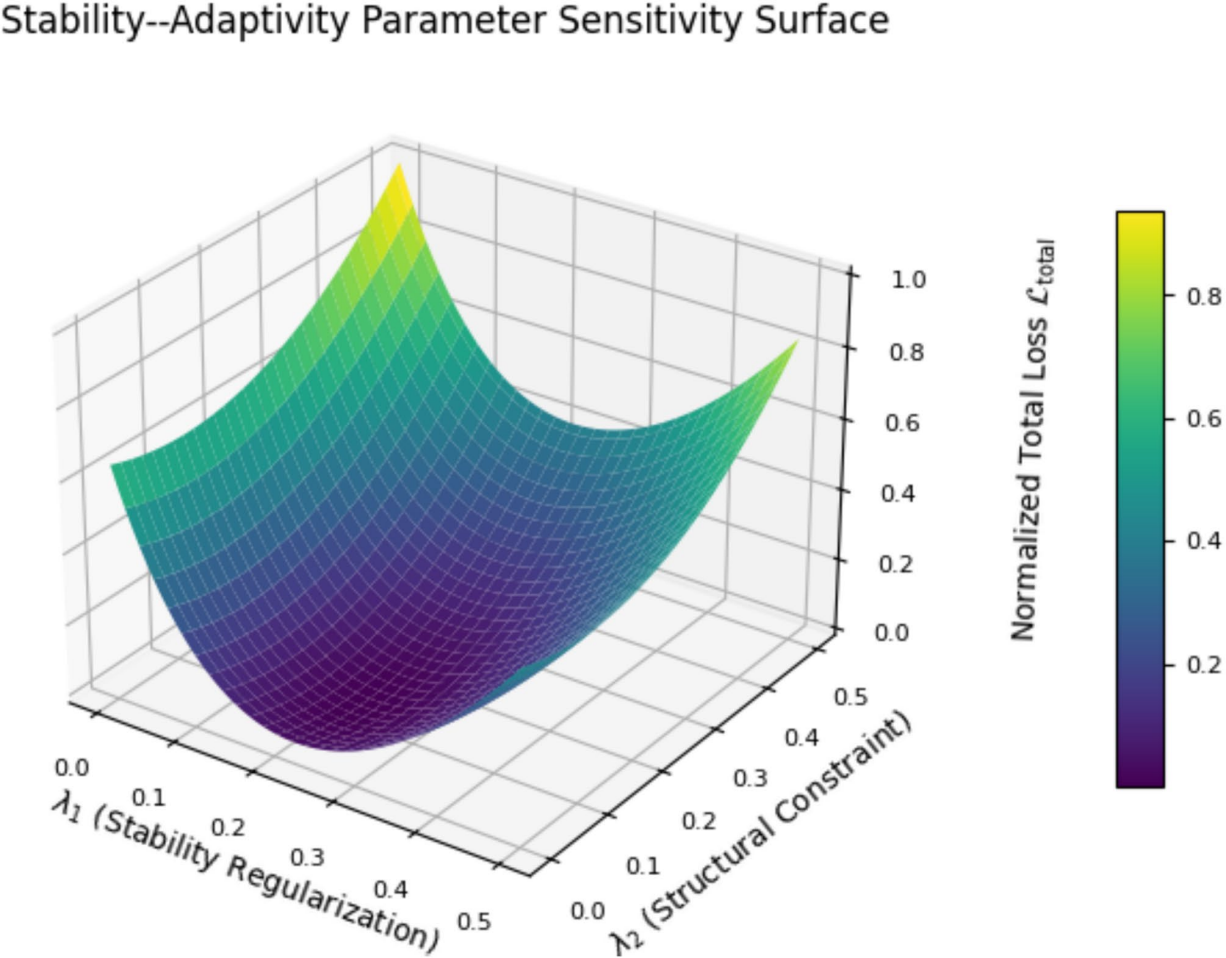
Stability–adaptivity parameter sensitivity surface of the proposed framework. The normalized total loss $\mathscr {L}_{\textrm{total}}$ is shown as a function of the stability regularization strength $\lambda _1$ and the structural constraint coefficient $\lambda _2$. A well-defined low-loss basin emerges at moderate regularization values, indicating a balanced regime that suppresses spurious temporal fluctuations without inducing oversmoothing or structural rigidity.

42 $$\begin{aligned} \mathscr {L}_{\textrm{total}}(\lambda _1, \lambda _2) = \mathscr {L}_{\textrm{task}} + \lambda _1 \, \mathscr {R}_{\textrm{stab}} + \lambda _2 \, \mathscr {R}_{\textrm{struct}}, \end{aligned}$$

Equation (42) defines the full optimization objective of the proposed framework, which explicitly balances task performance, temporal stability, and structural coherence. The coefficient $\lambda _1$ controls the strength of stability regularization that suppresses spurious temporal fluctuations, while $\lambda _2$ enforces structural consistency by penalizing low-modularity community assignments.

Importantly, the objective is not monotonic with respect to either coefficient. Small values of $\lambda _1$ lead to unstable solutions dominated by spurious temporal fluctuations, whereas excessively large $\lambda _1$ induces oversmoothing and suppresses meaningful community evolution. Similarly, overly strong structural constraints ($\lambda _2$ large) restrict adaptive reconfiguration and increase the total loss.

This interaction gives rise to a well-defined low-loss basin in the $(\lambda _1, \lambda _2)$ space, which is visualized in Fig. 6. The existence of such a basin indicates that the proposed framework admits a stable operating regime rather than relying on fragile hyperparameter tuning.

**Table 21:** Ablation study of the proposed stability components. Removing the memory anchor or adaptive weighting degrades NMI/ARI and increases temporal variation, showing that both components contribute to stable community tracking.

Variant	NMI	ARI	Q	TV
Full STR-GNN	$\mathbf {0.60 \pm 0.01}$	$\mathbf {0.56 \pm 0.01}$	*0.50 ± 0.01*	*0.21 ± 0.01*
w/o memory anchor	*0.56 ± 0.02*	*0.52 ± 0.02*	*0.49 ± 0.01*	*0.26 ± 0.02*
w/o adaptive weighting	*0.57 ± 0.02*	*0.53 ± 0.02*	*0.49 ± 0.01*	*0.24 ± 0.01*
uniform smoothing only	*0.54 ± 0.02*	*0.50 ± 0.02*	$\mathbf {0.52 \pm 0.01}$	$\mathbf {0.19 \pm 0.01}$
w/o stability regularizer	*0.55 ± 0.02*	*0.51 ± 0.02*	*0.49 ± 0.01*	*0.28 ± 0.02*

Significant values are in bold.

Table 21 isolates the contribution of the main stability components. Removing the memory anchor increases TV because the model no longer has a historical structural reference. Removing adaptive weighting also degrades performance, indicating that uniformly penalizing all nodes is less effective than assigning stronger regularization to unstable nodes. The uniform smoothing variant achieves the lowest TV and highest modularity, but it reduces NMI and ARI, confirming that excessive smoothing can suppress genuine community transitions. These results support the role of memory-guided and node-adaptive stability control in STR-GNN.

## Discussion

After conducting our research, we came to the conclusion that illusory temporal dynamics are a significant hurdle in the process of dynamic community detection on evolving graphs. In many temporal networks that are based on the actual world, successive snapshots frequently contain measurement noise, missing edges, or ephemeral interactions that do not accurately depict the evolution of the structure. The typical temporal graph neural networks, on the other hand, have a tendency to interpret these relatively minor fluctuations as meaningful signals. This, in turn, causes the representation oscillation to be magnified, which ultimately leads to misleading community shifts, even when the underlying structure remains fairly constant^34^.

This phenomenon suggests that temporal instability is not only a product of training but rather an intrinsic limitation of learning that is driven by snapshots. The majority of temporal GNNs do not have a global structural reference since they only employ the current graph and a short-range temporal dependence to update their node representations while they are being updated^35^. As a consequence, the update process may be dominated by high-frequency noise in the graph Laplacian spectrum, which results in rapid embedding fluctuations that appear as misleading community changes. When it comes to dynamic community detection, where the objective is to capture long-term structural change rather than instantaneous alterations, this kind of behavior is very detrimental^36^.

The results of our tests have shown that merely lowering the amount of temporal fluctuation is not enough. This is an important discovery. Strong temporal smoothing has the ability to successfully suppress oscillation; yet, at the same time, it reduces the model’s sensitivity to true structural changes^13^. When an excessive amount of smoothing is used, the embeddings of nodes become excessively coupled over time. This results in oversmoothing and a loss of community separability. Consequently, this provides an explanation for the non-monotonic relationship that was seen between temporal stability and detection performance in our studies using ablation and noise^37^. When it comes to robustness, moderate stabilization is beneficial, while excessive stabilization is detrimental because it decreases clustering accuracy and slows adaptation to actual structural shifts^38^.

By adding stability as a selected constraint rather than a global smoothing operation, the stability-aware architecture that has been suggested is able to overcome this problem. The model binds the current representation to a memory-informed structural reference that is created from several historical snapshots^39^. This is done rather than forcing subsequent embeddings to be identical to one another. This memory process supplies the model with long-range temporal context, which enables the model to differentiate between structural patterns that are durable and perturbations that are just temporary that occur. From a spectral point of view, the stability regularization eliminates high-frequency components that are connected with noise while maintaining low-frequency components that relate to the actual structure of the community^40^. As a result, the approach is able to strike a controlled equilibrium between adaptability and stability.

This design has a number of key implications, one of which is that stability ought to be seen as an explicit optimization aim rather than an implicit side effect of temporal aggregation^41^. Through the utilization of a parameter that can be adjusted, the model is able to manage the trade-off between structural consistency and responsiveness to change. This is accomplished by introducing stability regularization into the loss function^42^. Because of this, the framework is able to maintain its robustness even when subjected to noisy settings, while simultaneously tracking the evolution of the real community in streaming scenarios^43^.

Extensive studies conducted on a variety of dynamic graph benchmarks have demonstrated that the suggested method successfully lowers spurious temporal fluctuations without compromising the accuracy of community detection^44^. The improvement is especially noteworthy in noisy, streaming, and long-horizon scenarios, which are typical examples of situations in which conventional approaches frequently display highly unstable behavior^45^. Further evidence that the stability-aware method effectively suppresses spurious high-frequency dynamics is provided by quantitative evaluation that makes use of spectral energy ratio, temporal variation, and drift error.

spurious resistance should be considered a fundamental target in temporal graph learning, according to these data, which suggest that this should be recognized. In the future, dynamic community identification models should not rely exclusively on snapshot-level message forwarding; rather, they should explicitly take into consideration the relationship between noise amplification, temporal memory, and spectral stability^46^. Stability-aware modeling offers a potential route toward robust temporal representation learning, which is needed for reliable graph learning in settings that are noisy and constantly changing. The capacity to resist deceptive temporal dynamics is essential for effective graph learning^47^.

## Conclusions

We presented a memory-guided stability paradigm for dynamic community detection that explicitly mitigates illusory temporal dynamics in temporal graph neural networks. This framework was developed by us. In contrast to traditional temporal models, which are exclusively dependent on the sending of messages at the snapshot level, the method that has been suggested integrates a selected stability regularization that is directed by historical structural representations. It is important for successful temporal graph learning that the model be able to differentiate between high-frequency oscillations brought on by noise and true low-frequency community dynamics. This architecture makes it possible for the model to do so without any problems.

Instead than treating stability as an implicit consequence of temporal aggregation, the essential concept behind the technique that has been offered is to treat stability as an explicit optimization aim. The model anchors current node representations to permanent graph patterns by preserving a memory-based structural reference across many snapshots. At the same time, it allows for adaptive updates to be made in the event that actual structural changes take place. In terms of spectral analysis, the stability constraint is responsible for suppressing high-frequency components that are noisy in the embedding space, while at the same time maintaining low-frequency information that is consistent with the community. This selective filtering process eliminates the possibility of spurious temporal fluctuations without introducing an excessive amount of smoothness, which is a typical shortcoming in the approaches that are currently being used to create temporal graphs.

Extensive experiments conducted on a variety of dynamic graph benchmarks have demonstrated that the framework that has been described is capable of achieving a favorable balance between adaptability and stability on several occasions. When compared to conventional temporal GNN models, our strategy greatly reduces the amount of representation oscillation, enhances the accuracy of grouping, and maintains a greater level of temporal consistency even when the conditions are noisy and streaming. In situations where the graph sequence contains measurement noise, missing edges, or short-lived interactions, standard approaches frequently produce misleading community shifts. This advantage becomes more evident when the graph sequence comprises these attributes. It has been demonstrated through quantitative analysis that the proposed stability mechanism is capable of effectively suppressing spurious temporal fluctuations while simultaneously sustaining true structural evolution. This was accomplished by using temporal variation, spectral energy ratio, and drift error.

This paper presented STR-GNN, a stability-regularized extension for temporal GNN-based dynamic community detection. By combining historical structural memory with adaptive stability regularization, STR-GNN reduces spurious temporal fluctuations while preserving meaningful community evolution. Experiments under a unified streaming protocol show improved NMI and ARI compared with standard temporal GNN baselines, while the discussion of modularity and temporal variation highlights the trade-off between conservative smoothing and adaptive community tracking. Future work will extend the framework to continuous-time temporal graphs and larger-scale event-level settings. Temporal data are intrinsically noisy and frequently arrive in a streaming fashion in practical applications such as communication networks, citation graphs, and online social systems. These types of applications are examples of practical applications. It is possible for models that do not include stability control to overreact to transient changes, which might result in results that are not dependable for community detection. Through the incorporation of temporal memory, structural reference, and stability regularization into a cohesive framework, the memory-guided stability technique that has been developed offers a systematic approach to addressing this issue.

In a nutshell, the findings of this research demonstrate that expressive temporal modeling is not the only thing that is necessary for accurate dynamic community detection; explicit control of temporal instability is also required. This method offers robust temporal representation learning without sacrificing flexibility to genuine structural changes. This is accomplished through the introduction of a memory-guided stability constraint, which facilitates the learning process. It is suggested that spurious resistance should become a fundamental objective in future research on dynamic graph learning, particularly in noisy, long-horizon, and online scenarios. These results underscore the necessity of stability-aware design in temporal graph neural networks and propose that spurious resistance should become a component of the research.

## Future work

For the purpose of further enhancing stability-aware temporal graph learning, there are numerous potential routes for future study that require additional investigation^38,48^.

To begin, the existing system for maintaining stability makes use of a memory buffer of a set length in order to supply a historical structural reference during the process of temporal updates^49^. It is possible that a fixed memory horizon is not the best option for all dynamic graphs, despite the fact that this design is quite effective in suppressing short-term oscillations. The evolution of temporal networks in the real world frequently demonstrates multi-scale evolution, in which certain structural changes take place gradually over extended periods of time while others emerge fast^50^. Consequently, if the framework that has been proposed is extended to include support for adaptive or learnable memory horizons, it might be possible for the model to automatically modify its temporal context in accordance with the characteristics of the graph^51^. Utilizing techniques such as attention-based memory selection, hierarchical temporal aggregation, or learnable decay factors, for instance, may make it possible for the model to acquire long-term structural information while simultaneously maintaining its responsiveness to current changes. These kinds of techniques have the potential to further improve the equilibrium between adaptability and stability, particularly in situations involving streaming or long-horizon scenarios.

Second, the proposed formulation suppresses spurious temporal fluctuations in a roundabout way by means of stability regularization. It is important to note that this method does not directly manage the frequency components of temporal embeddings, despite the fact that it does reduce representation oscillation^4^. According to findings from recent research on temporal graph learning, noisy updates frequently correspond to high-frequency components in the graph spectral domain. On the other hand, actual community evolution is typically related with low-frequency structural variation. It is therefore possible that the incorporation of explicit frequency-aware objectives will allow a more accurate control over temporal stability. Spectral filtering, frequency-domain regularization, or Laplacian-based energy restrictions are some examples of techniques that could be utilized to directly penalize high-frequency oscillations while also preserving relevant structural information. This type of frequency-aware learning has the potential to further minimize spurious temporal fluctuations and increase robustness in settings that are noisy or partially observable^46,47^.

The third point is that although the current work is centered on dynamic community detection, the problem of learning temporal representations that are resistant to spurious is a more general issue that arises in a variety of graph-based operations. For the purposes of temporal link prediction, anomaly detection, change-point detection, and influence analysis, it is necessary to have a modeling of developing graph structure that is both stable and adaptable. In the context of these applications, an excessive amount of temporal fluctuation may result in false alarms, predictions that are not dependable, or decision-making that is unstable. It is possible that a unified solution for robust temporal graph learning might be provided by extending the stability-aware framework that has been suggested to accommodate these tasks. The integration of stability requirements with task-specific objectives, in particular, has the potential to provide more reliable inference in situations that involve streaming, large-scale networks, and noisy environments.

When taken as a whole, these tendencies indicate that future research should progress toward stability-aware and frequency-aware temporal graph learning. In this approach, spurious resistance would become a basic design principle rather than an additional regularization technique. These kinds of advancements have the potential to greatly enhance the dependability of dynamic graph neural networks in applications that take place in the real world.

## Data Availability

The datasets used and/or analysed during the current study available from the corresponding author on reasonable request.
